# Unified Description of Ultrafast Excited State Decay Processes in Epigenetic Deoxycytidine Derivatives

**DOI:** 10.1021/acs.jpclett.1c02909

**Published:** 2021-11-08

**Authors:** Piotr Kabaciński, Marco Romanelli, Eveliina Ponkkonen, Vishal Kumar Jaiswal, Thomas Carell, Marco Garavelli, Giulio Cerullo, Irene Conti

**Affiliations:** † IFN-CNR, Dipartimento di Fisica, Politecnico di Milano, Piazza Leonardo da Vinci 32, I-20133 Milano, Italy; ‡ Dipartimento di Chimica Industriale, Università degli Studi di Bologna, Viale del Risorgimento 4, I-40136 Bologna, Italy; § Department of Chemistry, Ludwig-Maximilians-Universität München, Butenandtstrasse 5-13, Munich 81377, Germany

## Abstract

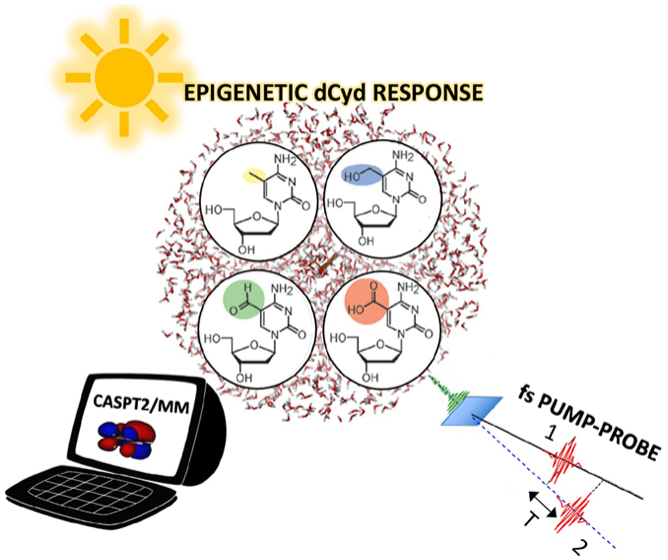

Epigenetic DNA modifications play a fundamental role in modulating gene expression and regulating cellular and developmental biological processes, thereby forming a second layer of information in DNA. The epigenetic 2′-deoxycytidine modification 5-methyl-2′-deoxycytidine, together with its enzymatic oxidation products (5-hydroxymethyl-2′-deoxycytidine, 5-formyl-2′-deoxycytidine, and 5-carboxyl-2′-deoxycytidine), are closely related to deactivation and reactivation of DNA transcription. Here, we combine sub-30-fs transient absorption spectroscopy with high-level correlated multiconfigurational CASPT2/MM computational methods, explicitly including the solvent, to obtain a unified picture of the photophysics of deoxycytidine-derived epigenetic DNA nucleosides. We assign all the observed time constants and identify the excited state relaxation pathways, including the competition of intersystem crossing and internal conversion for 5-formyl-2′-deoxycytidine and ballistic decay to the ground state for 5-carboxy-2′-deoxycytidine. Our work contributes to shed light on the role of epigenetic derivatives in DNA photodamage as well as on their possible therapeutic use.

Epigenetics, which is the study of heritable phenotype modifications that do not involve alterations in the genotype, is becoming a more and more important field of research, aiming to explain how living organisms adapt to external stimuli.^1^ Methylation of 2′-deoxycytidine (dC) at the C_5_ position of the nucleobase can lead to transcriptional silencing of the corresponding gene in certain genomic regions.^2^ 5-Methyl-2′-deoxycytidine (mdC) is a prevailing epigenetic modification that plays important roles in modulating gene expression and developmental processes, and its dysregulation may cause severe diseases, including cancer.^1,3−6^ Demethylation of mdC back to dC reactivates the transcription of these genes; however, the process behind this demethylation remains not yet fully understood. A decade ago, 5-hydroxymethyl-2′-deoxycytidine (hmdC),^7^ 5-formyl-2′-deoxycytidine (fdC),^8^ and 5-carboxyl-2′-deoxycytidine (cadC)^9^ were detected as additional epigenetic elements in DNA. Furthermore, it was shown that these modified dC bases are formed from mdC via consecutive oxidation reactions catalyzed by 10–11 translocation enzymes.^9,10^ These oxidized mdC derivatives are considered to form a second layer of information and to be a part of an active DNA demethylation process that potentially regulates the concentration and pattern of epigenetic markers in mammalian cells.^3−6,10^

Epigenetic dC derivatives might affect the efficient and ultrafast nonradiative excited state (ES) deactivation channels of the canonical nucleosides, which safely dissipate the absorbed light energy, possibly leading to more complex scenarios of DNA photoprotection and photodamage.^11−15^ According to quantum mechanics/molecular mechanics (QM/MM)^16^ calculations at the CASPT2^17,18^ level, the classical cytidine dC and the most common epigenetic methylated form (mdC) show different energy barriers of ∼0.18 and ∼0.27 eV, respectively, along the same decay pathway, driving the lowest ππ* ES to the “ethylene-like” conical intersection (CI)^19−22^ with the ground state (S_0_). This difference justifies the significantly longer lifetime of the epigenetic derivative (6.8 ps) with respect to the parent compound (1.1 ps),^23,24^ observed with femtosecond transient absorption (TA) spectroscopy, which makes mdC more prone to photodamage events. In addition, dark nπ* ESs, which are thought to play a role in the long-living component of the observed TA signal for water-solvated dC, are predicted to be destabilized in mdC and thus not to be involved in the relaxation of the lowest ππ* state.^23,24^ Recent experimental^25^ studies, supported by CASSCF or TDDFT computations,^26,27^ showed that while the photophysics of hmdC substantially resembles that of mdC, the ES relaxation pathways of fdC and cadC are remarkably different. In fdC, there is experimental evidence of an efficient ultrafast intersystem crossing (ISC) that leads to the population of the lowest triplet state, accounting for a long-living component of the TA signal, whereas water-solvated cadC shows a subpicosecond (840 fs) ES decay.^25^ The photophysical processes underlying these very different excited state dynamics aroused increasing interest, becoming a current matter of debate and giving rise to contradictory hypotheses on the decay mechanisms at play.^25−27^

Here, we aim to provide a unified and coherent description of the complex ES decay pathways of the epigenetic cytidines through a comprehensive experimental and theoretical investigation. On the experimental side, we perform ultrafast TA spectroscopy with state-of-the-art sub-30-fs temporal resolution to follow the rapid evolution of the photoexcited wave packet on the excited state potential energy surface (PES) and broad spectral coverage in the 1.9–3.9 eV range to identify all the photoinduced signals, including the previously unexplored UV region.^24,25^ Thanks to the high sensitivity of our TA apparatus, experiments are performed at low fluences, at which no formation of solvated electrons is observed, permitting the correct assignment of the TA signals. On the computational side, we employ a hybrid SS-CASPT2/MM scheme accounting for multireference dynamically correlated energies and gradients on all the epigenetic cytidines simultaneously, including the sugar moiety, and considering explicitly the water solvent along with hydrogen bonds (instead of an implicit continuum as in the polarizable continuum model^26−28^), which is necessary for a realistic description of the ES dynamics and of the spectroscopic signals, as previously demonstrated.^11,23,29,30^ We systematically map the major decay pathways (singlet and triplet, ππ* and nπ* states) based on the minimum energy paths involving all the characterized critical points and CIs driving the different photoprocesses. Eventually, this study allows new light to be shed on previously detected deactivation channels and reveals new ones falling in the so far uncharted sub-500-fs regime.

Figure 1a shows the chemical structures of the four epigenetic nucleosides, obtained from dC by substitution at the C_5_ position of the nucleobase. The corresponding absorption spectra, shown in Figure 1b, are dominated by an intense band spanning 4.1–4.6 eV due to the ππ* transition of the aromatic ring, similar to the canonical nucleosides.

**Figure 1:**
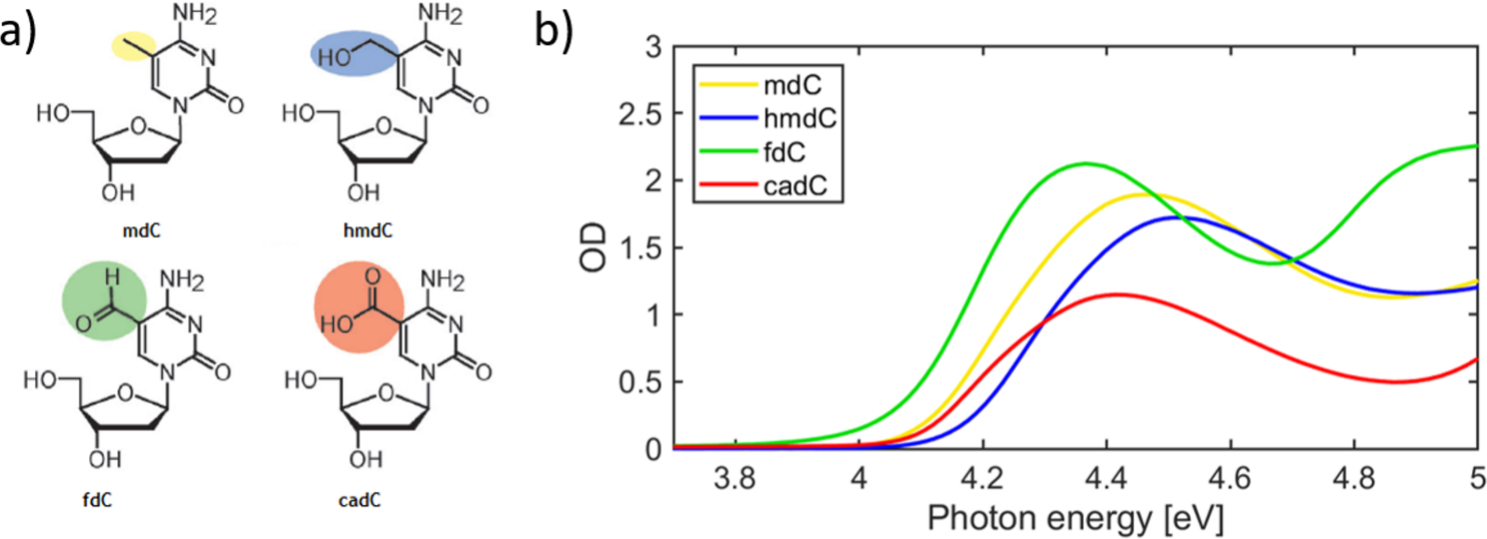
(a) Epigenetic derivatives of 2′-deoxycytidine studied and (b) their linear absorption spectra. The epigenetic modifications involve the C_5_ position of cytidine. The cadC nucleoside is in the anionic form in our simulations, because it is the stable species at neutral pH.

Figure 2a plots the differential absorption (ΔA) spectrum, as a function of pump–probe delay (up to 1 ps) and probe photon energy, for mdC following photoexcitation by a sub-20-fs pulse at 4.35 eV, which populates the lowest ππ_1_* bright ES. At early times, we observe a negative band (Figure 2c, blue line), peaking at 3.68 eV, assigned to stimulated emission (SE) from the bright ππ_1_* state, together with a positive photoinduced absorption (PA) band. Both the SE and PA bands undergo a rapid partial decay on the ∼100 fs time scale, showing a subsequent SE red-shift to 3.54 eV, as illustrated in Figure 2c (purple line), which displays the evolution associated spectra (EAS) obtained by global analysis of the TA data. Subsequently, the spectrum decays further in 1 ps while still shifting to the red, followed by a longer 4.3 ps decay into a low-intensity spectrum lacking any SE signals.

**Figure 2:**
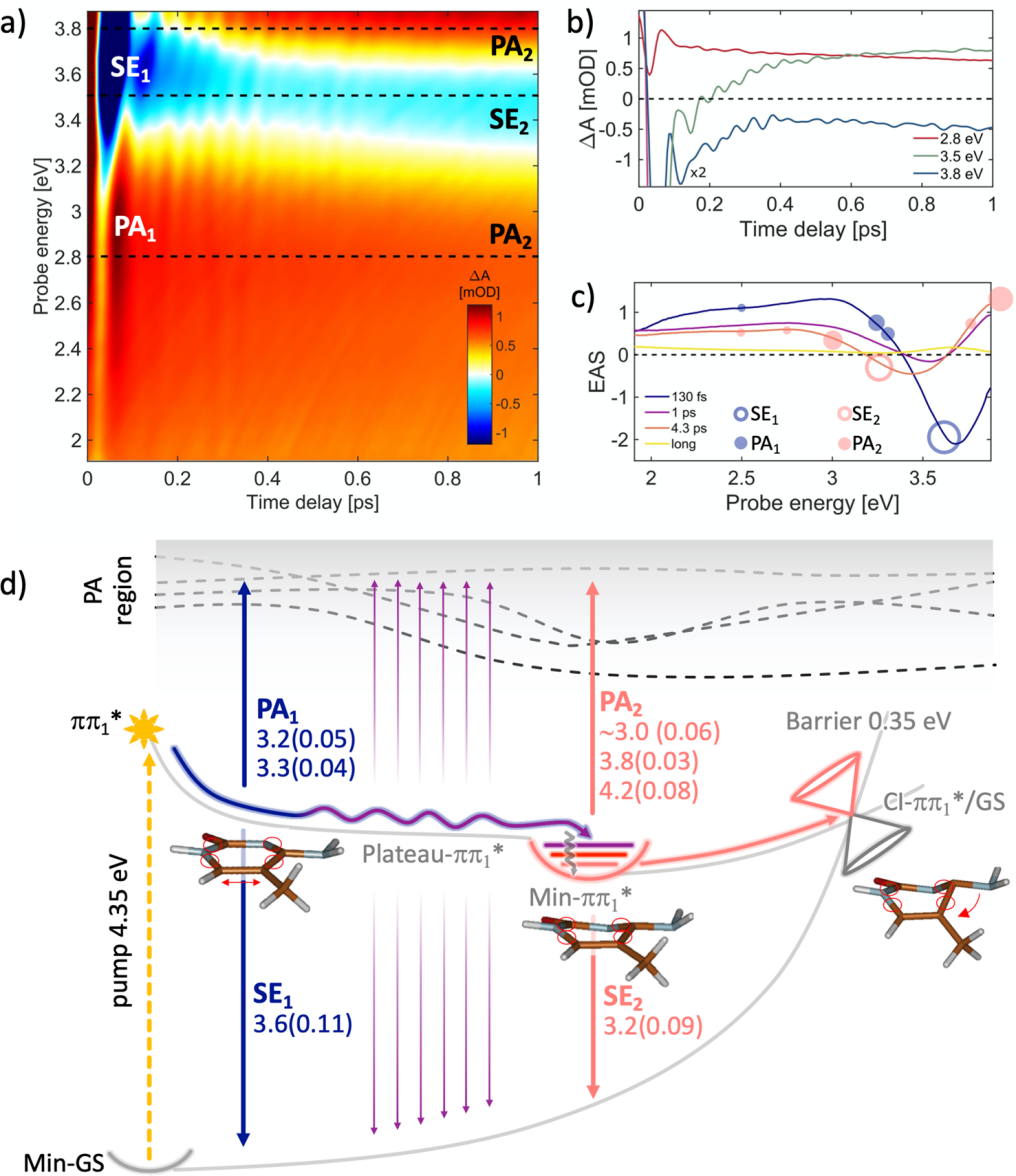
(a) ΔA map of mdC in water solution recorded with pulse polarizations at the magic angle. (b) Dynamics at selected probe photon energies marked with dashed lines in panel a. (c) EAS with the corresponding time constants: 130 fs (blue curve) is the time needed to relax from the FC to the Plateau-ππ_1_* flat region (see calculated paths in panel d), and 1 ps (purple curve) is the time needed to fully relax from the Plateau-ππ_1_* region to the Min-ππ_1_* (wavy purple line, panel d). The 4.3 ps time constant (pink curve in panel c) relates to the ππ_1_* → GS decay process (CI-ππ_1_*/GS, panel d), involving a 0.35 eV barrier. The yellow line corresponds to long-lived products probably due to other minor decay paths. Empty and full circles correspond to the calculated SE and PA energy values, respectively, and the circle dimensions are proportional to the computed oscillator strength values (documented in panel d). (d) Schematic decay paths of mdC, calculated at CASPT2/MM level (details in the SI section). Relaxation routes and SE/PA colors arrows are matching with the line colors of time constants in panel c. Oscillator strengths are reported in brackets. Critical point energies are in Figure S6. Molecular optimized structures refer to the QM region only.

Experimental TA data are consistent with the ES deactivation scenario derived from QM/MM calculations, which is summarized in Figure 2d. The calculations reveal an ultrafast relaxation from the Franck–Condon (FC) region of the ππ_1_* (S_1_) state toward a flat region of the ES PES, characterized by low forces acting on the system (Plateau-ππ_1_*, Figure 2d), where the computed SE band at 3.6 eV with an oscillator strength (OS) of 0.11 (SE_1_ in Figure 2d) matches the experimental SE band observed immediately after excitation (empty blue circle in Figure 2c).

Calculations also reproduce the peaks of the PA spectrum (full blue circles in Figure 2c) observed in the visible at early times (PA_1_), when the system came out of the Franck–Condon region and reached the Plateau-ππ_1_* region (vertical blue arrows in Figure 2d). The observed decrease in intensity and red-shift of the SE band seen in the ∼100 fs time scale (change from blue to purple EAS in Figure 2c) is assigned to molecules moving and oscillating across the flat Plateau-ππ_1_* region (see oscillating purple line in Figure 2d) until fully relaxing to the minimum (Min-ππ_1_*) within ∼1 ps. Critical point energies are reported in Figure S6 in the Supporting Information (SI). Here, the system resides for a longer time, due to the barrier that needs to be overcome to decay to the GS (4.3 ps time constant, pink line in Figure 2d). Note that the colors of the lines and arrows in Figure 2d, indicating the different decay processes, match those of the EAS curves of Figure 2c. The computed SE (SE_2_ in Figure 2d) from the Min-ππ_1_* structure is at 3.2 eV (OS 0.09) and is red-shifted compared to SE_1_. The overlap of the SE_2_ with the positive PA_2_ band, predicted from Min-ππ_1_* at 3.0 eV (OS 0.06, pink arrow in Figure 2d), could account for the observed reduction of the intensity and the blue-shift of the experimental SE peak with respect to the computed value (SE_2_, pink empty circle in Figure 2c). Moreover, starting from the ES minimum (Min-ππ_1_*), we also calculated PA_2_ signals at 3.83 eV (OS 0.03) and 4.22 eV (OS 0.08) that match with the experimental PA band observed in that spectral region (additional pink full circles in Figure 2c). The 4.3 ps decay of the SE_2_ signal coming from Min-ππ_1_* together with the corresponding PA_2_ (pink line, Figure 2c) is consistent with the fact that the lowest-lying CI between ππ_1_* and S_0_ features an energy barrier from the Min-ππ_1_* of ∼0.35 eV. Following the internal conversion (IC) to S_0_, which is the dominant decay pathway, a residual weak PA spectrum remains (yellow line) lasting longer than the probed time window (30 ps) because of other possible minor decay routes.

The calculations associate the first PA_1_ and SE_1_ signals (corresponding to the so far not observed shortest decay time constant on the order of 100 fs) to still planar structures just relaxed out of the FC region, beginning to distort along the ring-puckering coordinate in the flat Plateau-ππ_1_* region, leading to Min-ππ_1_* within the second time constant of ∼1 ps. The corresponding structural changes are illustrated in Figure 2d. It is worth noting that although our optimized ππ_1_*/GS CI does not exactly reproduce the structure of the “ethene-like” CI reported previously by Martinéz-Fernandéz et al.,^23^ they both show comparable access energy barriers (0.35 and 0.3 eV, respectively), but the CI documented in Figure 2d should be more easily accessible, as it lies exactly along the reaction coordinate that coherently connects the planar structure to the crossing, passing through the Min-ππ_1_*. Structural details about the computed ππ_1_*/GS CI for mdC are reported in the SI section, including the Cartesian coordinates.

We also investigated the photophysics of mdC when the higher-energy bright state S_2_ (ππ_2_*) is populated: a sudden decay to S_1_(ππ_1_*) is predicted owing to a crossing with the S_1_ state nearby to the S_2_ FC region, thus showing that the ππ_1_* state collects also the ππ_2_* population. In addition, dark states (nπ*) are destabilized compared to bright states in water solution, and therefore, they are not involved in the ES decay pathway when pumping at 4.35 eV (see calculated energies for the corresponding vertical and critical points shown in Figure S6).

HmdC exhibits photophysics very similar to that of mdC upon UV photoexcitation at 4.35 eV. Both ultrafast TA spectra and the calculated decay pathways strongly resemble those of mdC (see Figure S7). Following the photoexcitation into S_1_ (ππ_1_*), the population initially decays toward the Plateau-ππ_1_* region with a 160 fs time constant, and moving along the plateau region, it reaches the lowest minimum (Min-ππ_1_*) with a 735 fs time constant, again showing a red-shift of the SE spectrum. This behavior, already observed for the methylated compound, can be rationalized in the same way, including the previously undetected fast decay with a 160 fs time constant and similarly assigned to the initial planar relaxation, before population of the ring-puckering mode. A comparable energy barrier (∼0.30 eV) has to be passed to reach the crossing point of ππ_1_* with the GS, which presents very similar molecular distortions (see molecular structures in Figure S7) to those found for mdC, through which the molecule decays in 4.6 ps. The similarity of the photophysics is confirmed by the resemblance of the TA spectra and time constants of mdC and hmdC (Figures 2 and S7, respectively). The difference between the two TA maps is mostly due to the higher intensity of the PA bands relative to the SE for hmdC. Once again, computations reveal that photoexcitation of the second bright ππ_2_* state (S_2_) immediately leads to a crossing with ππ_1_* (S_1_), and no dark states (including all the low-lying nπ* states) seem to be involved in the ES decay pathway upon pumping at 4.35 eV (see the computed critical points in Figure S8).

FdC shows a very different decay scenario, compared to the other epigenetic cytidine derivatives (Figure 3). This is due to the presence of two almost isoenergetic low-lying ESs in the FC region that were not predicted before,^26,27^ namely S_1_ (ππ_1_*) and S_2_ (nπ*) (Figure 3d, yellow star and circle, respectively). This immediately leads to branching of the ES population. Only a CASPT2-correlated method combined with explicit solvent interactions predicts the ππ*/nπ* degeneracy in the FC vertical region (see also Figure S4): upon pumping at 4.35 eV, the bright ππ* S_1_ state (Figure 3d, right part) is mainly populated, and its simulated spectral signatures (blue arrows in Figure 3d) match well with the experimentally observed SE and PA_1_ signals shown as blue circles in Figure 3c. Simultaneously, the nπ* state is partially populated, contributing with its positive PA signals in the 3.3/3.9 eV regions (see Min-nπ* PA_2_ signals in Figure 3d). For the ππ_1_* state, an ultrafast relaxation pathway leads to the Plateau-ππ_1_* (right side of Figure 3d), a planar region of the ππ_1_* state that spans a progressively decreasing S_1_–S_0_ energy gap (from ∼3.30 to ∼1.0 eV) due to the corresponding increase in the GS energy. Here, the structure undergoes large distortions along the “ethene-like” coordinate: starting from a quite planar geometry, a large torsion around the C_2_N_1_–C_6_C_5_ angle (until ∼54°) and a C_5_ formyl out of plane bending (until ∼100°) take place (see molecular structures on top of the ππ_1_* decay path, Figure 3d). By continuing the optimization along S_1_, one finds a low-lying CI with the GS (CI-ππ_1_*/GS) that presents no access energy barrier from the plateau region, thus suggesting an ultrafast decay pathway (critical point energies in Figure S9). Indeed, the SE signal observed just after the excitation (blue line in Figure 3c) disappears with a 130 fs time constant: the purple line (Figure 3c) possibly represents a later stage of the evolution on the Plateau-ππ_1_* region where the ππ*-GS energy gap is reduced until the wavepacket decays to the GS in 345 fs, supporting our proposed mechanism (purple ππ_1_* wavy and GS arrows in Figure 3d).

**Figure 3:**
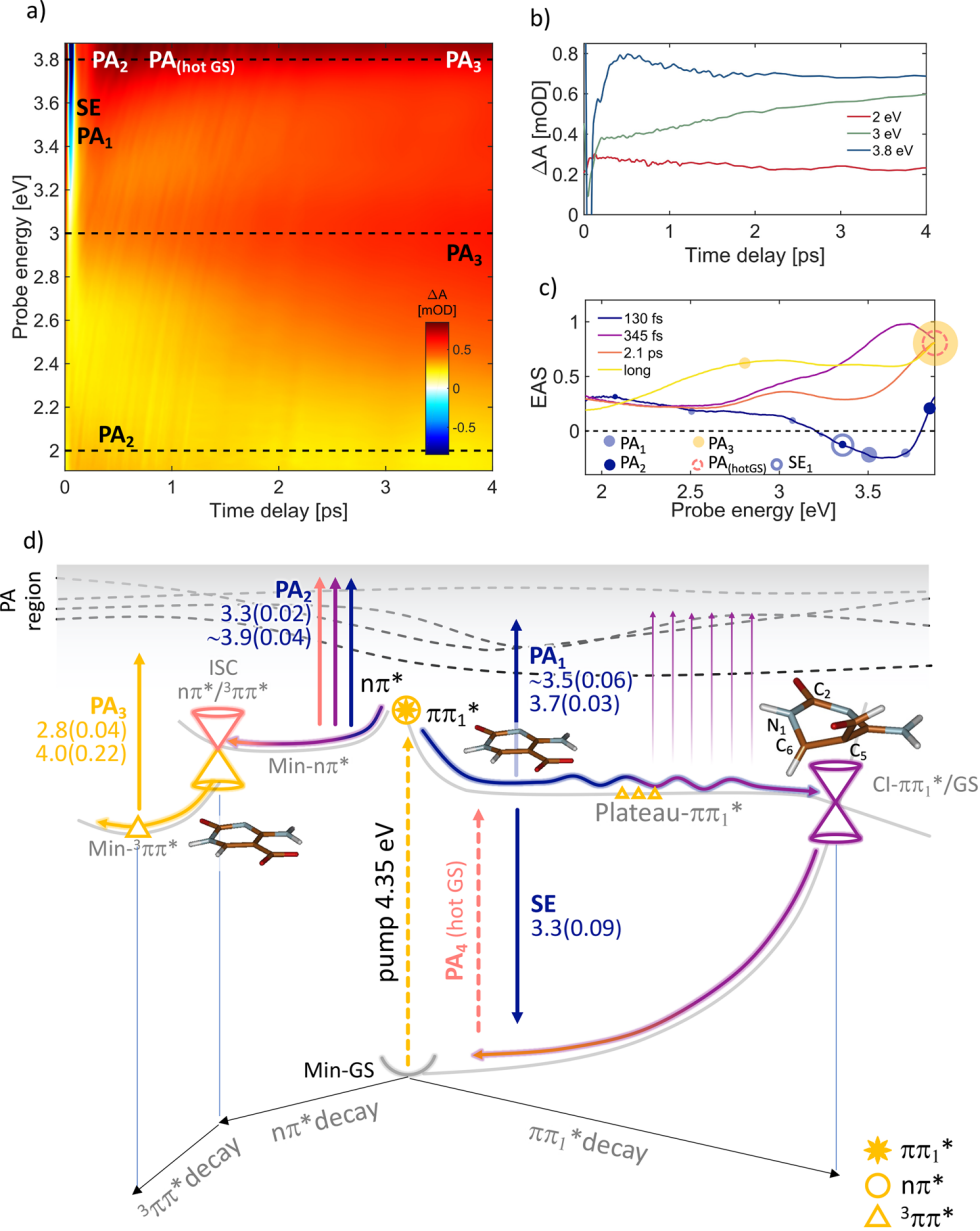
(a) ΔA map of fdC in water solution recorded with pulse polarizations at the magic angle. (b) Dynamics at selected probe energies (eV) marked with dashed lines on panel a. (c) EAS with the corresponding time constants: 130 fs (blue curve) is the time needed to relax from the FC to the Plateau-ππ_1_* flat region (calculated paths in d panel, right side) and simultaneously to the Min-nπ* (left side, panel d), 345 fs (purple curve) corresponds to the time to decay on the GS from the Plateau-ππ_1_* through CI-ππ_1_*-GS (wavy purple line, panel d), and the 2.1 ps time constant (pink curve, panel c) relates to the ISC ^1^nπ*/^3^ππ* decay process (panel d, left side). The yellow line corresponds to long-living triplet state minimum (Min-^3^ππ*). Empty and full circles correspond to the calculated SE and PA energy values, respectively, and the circle dimensions are proportional to the computed oscillator strength values (documented in panel d). (d) Schematic decay paths of fdC, calculated at the CASPT2/MM level (details in the SI section). Relaxation routes and SE/PA colors arrows are matching with the line colors of time constants in panel c. Oscillator strengths are reported in brackets. Critical point energies are in Figure S9. Molecular optimized structures refer to the QM region only.

While two different fdC conformers may exist in water,^27^ only the one lacking an intramolecular hydrogen bond between the amino and the formyl groups was discussed in this study (*anti* isomer) while neglecting the conformer where the formyl carbonyl and the amino group are bridged through an intramolecular N–H···:O bond (*syn*), which possibly could induce molecular restraints. The *anti* choice was taken, because this conformer is the one that, we believe, is more relevant for the ultrafast sub-400-fs photoinduced dynamics observed in this study (see the SI, section 4.1, for a detailed discussion). Moreover, very recent time-resolved IR experiments and TDDFT calculations^27^ show that out of plane motions (described for the *anti* conformer) are indeed populated regardless of the fdC conformer.

On the other hand, the aforementioned S_2_ dark state (nπ*) is almost isoenergetic with the ππ_1_* (S_1_) at the FC point: surface crossing between these two states leads to population of the dark nπ* singlet state (S_2_) at early times through IC, already within 130 fs (blue line). In addition, our vertical calculations do not consider the vibrational degrees of freedom of the molecule, thus neglecting the plausible contribution to the S_2_ OS coming from distorted molecular geometries. The simultaneous population of the ππ_1_* and nπ* states could be supported by the weaker fdC steady state fluorescence spectrum^27^ as compared with mdC,^22^ which instead populates just the bright ππ_1_* state, because the nπ* state lies at higher energies (see Figure S6). Following the dark nπ* state, the optimization of S_2_ leads to a minimum (Min-nπ*, Figure 3d), where the computed PA_2_ values (3.3 and 3.9 eV in Figure 3d and dark blue circles in Figure 3c) contribute to the first three time constants (blue, purple, and pink lines, Figure 3c), because its decay via ISC processes could require picosecond time scales, as also supported by recent time-resolved mid-IR spectroscopy experiments.^27^ The experimental spectrum also contains a contribution from the hot GS PA, following ultrafast decay through CI-ππ_1_*/GS. Hot GS relaxation is a process typically falling in the picosecond time range (pink line, dashed circle in Figure 3c and pink dashed arrow in Figure 3d).

A crucial characteristic of the nπ* relaxation path is that at the minimum geometry (Min-nπ*) the lowest ^3^ππ* triplet excited state (gray triangle in Figure S9) is close in energy to the singlet nπ*, allowing an ISC process that results in an efficient population of the T_1_ triplet excited state minimum (Min-^3^ππ*, yellow triangle in Figure 3d). The decay of the nπ* spectrum in 2.1 ps (pink line in Figure 3c) into the remaining long-lived spectrum (yellow line Figure 3c) is attributed to the population of this lowest triplet state, which survives for times much longer than our probing window. In support of this mechanism, the PA_3_ values computed on top of the triplet minimum (yellow arrow, 2.8 and 4.0 eV) show good agreement with the experimental peak around 2.84 eV (growing in the pink and clearly recognizable in the long-living yellow line, Figure 3c) as well as with the more intense UV-shifted signal at 4.0 eV (better recognizable in the DUV probe spectrum in Figure S11), which exhibits a higher OS compared to the previous transition (0.22 vs 0.04), thus justifying the strong absorption tail on the blue edge of the spectrum. The high triplet quantum yield^25,31^ could also be attributed to a further minor contribution coming from the Plateau-ππ* region, in which the triplet is isoenergetic to the bright state (yellow triangles on the right side of Figure 3d).

The combination of sub-30-fs TA spectroscopy and state-of-the-art CASPT2/MM calculations thus enables one to derive a detailed picture of the different photoinduced processes in fdC, assign the observed decay time constants, and understand the pathway leading to population of the triplet state. The ππ* ↔ nπ* IC in the FC region, later leading to the ISC process, and the ultrafast barrierless ππ_1_* → S_0_ decay path are both fundamental and previously unpredicted excited state deactivation processes.^25−27^

Finally, and notably, the photophysics of cadC upon pumping at 4.35 eV is quite different from that of the previous derivative. Surprisingly, there is no evidence of triplet formation and the experimental signal shows an ultrafast relaxation that can be assigned to direct decay from the ππ_1_* ES to the GS. In this molecule, unlike the fdC derivative, computations do not identify any low-lying dark state that is isoenergetic with ππ_1_* (S_1_) in the FC region. The experimental TA map (Figure 4a) and dynamics (Figure 4b) are dominated by SE (at 3.68 eV) and broad PA (at 2.33 eV) bands at early times that shift and decay on the ∼100 fs time scale to give rise to a PA band above 3.8 eV together with small remaining intensity in the shifted PA (near IR region), which in turn decays on the picosecond time scale. The corresponding EAS (Figure 4c) decays with the very fast 130 fs time constant, giving rise to a characteristic spectrum of hot GS PA decaying with a 960 fs time constant (previously incorrectly assigned to the ππ* → S_0_ decay^25^). By optimizing the lowest ππ_1_* state, we found once again a flat region of the PES (Plateau-ππ_1_*) where the SE signal (SE_1_ at 3.7 eV in Figure 4d) matches the short-living experimental signal (130 fs, blue empty circle Figure 4c). These data indicate that the ππ_1_* → GS decay process is ultrafast (with a 130 fs time constant, blue line in Figure 4c), leading straight to the CI in a ballistic fashion (CI-ππ_1_*/GS, Figure 4d), differently from the mdc and hm-dC derivatives described above, where the flatter region of the S_1_ PES, leading to the Min-ππ_1_*, and the energy barrier work as a trap (Figures 2 and S7).

**Figure 4:**
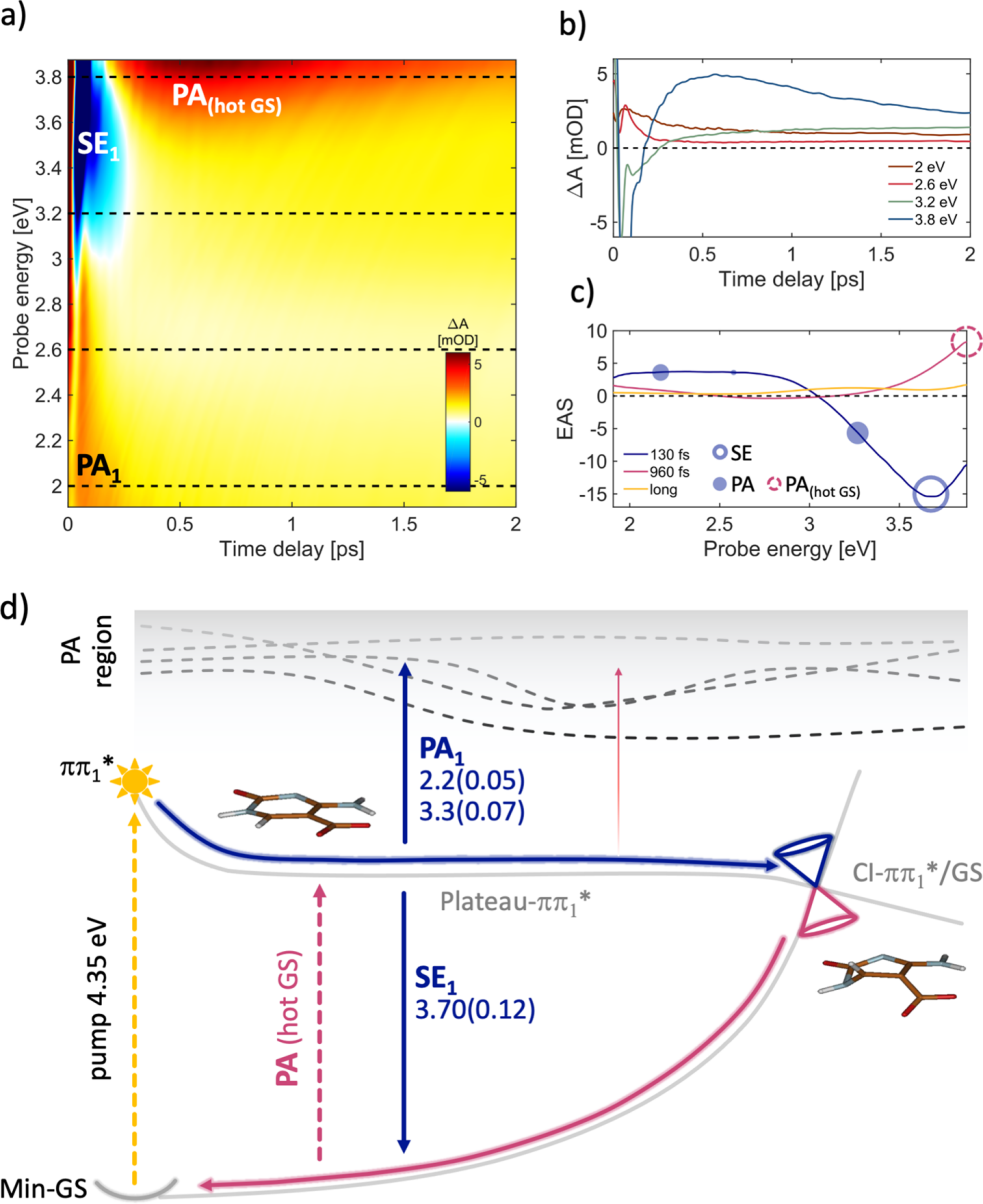
(a) ΔA map of cadC in water solution recorded with parallel pulse polarizations. (b) Dynamics at selected probe energies (eV) marked with dashed lines on panel a. (c) EAS with the corresponding time constants: 130 fs (blue curve) is the time needed to ballistically relax from the FC to the ππ_1_*/GS CI (see calculated blue paths in panel d), and 960 fs pink curve signals correspond to the hot GS repopulation, following the ultrafast IC (calculated decay path in panel d). The yellow line corresponds to long-lived products probably due to other minor decay paths. Empty and full circles correspond to the calculated SE and PA energy values, respectively, and the circle dimensions are proportional to the computed OS values (documented in panel d). (d) Schematic decay paths of cadC, calculated at CASPT2/MM level (details in the SI section). Relaxation routes and SE/PA colors arrows are matching with the line colors of time constants in panel c. Oscillator strengths are reported in brackets. Critical point energies are in Figure S10. Molecular optimized structures refer to the QM region only.

On the other hand, we attribute the red tail of the second EAS spectrum (pink line in Figure 4c) to the residual population remaining trapped on the Plateau-ππ_1_* (similarly to the barrierless ππ_1_* ultrafast evolution of the fdC), where the SE is almost negligible, showing only a very weak tail around 2.9 eV. While a significant part of the ES population decays on an ultrafast time scale through this ππ_1_*→ GS IC channel, there is also a low-intensity PA signal left for times longer than 30 ps (Figure 4c, yellow) that might be due to other minor decay pathways.

In conclusion, our joint experimental/computational study provides a comprehensive picture of the ES dynamics of all four epigenetic 2′-deoxycytidine nucleosides. By combining ultrafast TA spectroscopy with sub-30-fs temporal resolution with CASPT2/MM computations explicitly considering the water solvent, we have shown how the different chemical modifications dramatically affect the de-excitation pathways. By replacing the hydrogen atom at the fifth position of the pyrimidine ring with a methyl or hydroxymethyl group, the ultrafast ES decay along the S_1_ PES, as compared to the parent molecule,^23^ is slowed down due to an increased energy barrier to reach the ππ_1_*/GS CI (0.35 or 0.30 eV, respectively), compared to the standard nucleoside (0.18 eV^23^). Indeed, the experimentally recorded SE signal, which provides an unambiguous spectroscopic fingerprint of the ππ_1_* state, decays in ∼4 ps for the methylated and hydroxymethylated derivatives in contrast with the typical subpicosecond decay of 2′-deoxycytidine. For these molecules, we also observe an initial ultrafast decay (∼130–160 fs time constant), associated with the fast relaxation out of the FC region. Moreover, the low-lying dark states that are thought to be involved in the excited state relaxation path of water-solvated 2′-deoxycytidine are destabilized in these derivatives^24^ and are therefore not involved in the main relaxation pathway.

Substitution of a C_5_ hydrogen of the cytosine ring by a formyl group significantly changes the ES dynamics. We first identify a dark nπ* state, which is nearly energetically degenerate with the bright ππ* state in the FC region, that can be thus immediately populated, eventually enabling an ultrafast 2 ps ISC process from the nπ* state minimum, which gives rise to a long-lived lowest triplet state, in agreement with previous studies.^25^ We also characterize a new additional and simultaneous ππ_1_* → S_0_ ultrafast decay pathway, leading directly back to the ground state.

Finally, the carboxyl derivative displays the shortest, and previously uncharted, ES lifetime among all epigenetic dC nucleosides, dominated by the ultrafast decay of the lowest ππ_1_* (S_1_) to the GS with a 130 fs time constant due to a ballistic wavepacket motion toward a low-lying barrierless CI. The 960 fs time constant, previously assigned to the ππ_1_* → GS IC process, is now attributed to GS vibrational cooling.^25^

This work represents an important step toward a comprehensive picture of the intricate photophysical decay mechanisms of epigenetic dC derivatives in the biologically relevant aqueous environment, which display a dramatic sensitivity to C5 substitutions. Our results help to elucidate their role in the incidence of DNA photodamage, promoted by either longer excited state lifetimes or population of the triplet states, which leads to the generation of destructive singlet oxygen and makes the epigenetic derivatives more reactive or, on the other hand, possibly suitable in medical applications as phototherapeutic agents.

## Supporting Information Available

Computational details; 5-methyl-2′-deoxycytidine: critical points and conical intersection calculations; benchmark calculations; 5-hydroxymethyl-2′-deoxycytidine: time-resolved spectra, decay paths, critical points, and conical intersection calculations; 5-formyl-2′-deoxycytidine: critical points and conical intersection calculations; 5-formyl-2′-deoxycytidine: formyl in *syn* and *anti* conformation; 5-carboxyl-2′-deoxycytidine: critical points and conical intersection calculations; chemical synthesis; sample preparation; TA setup description; DUV probe measurement of fdC; parallel polarizations for mdC and hmdC and magic angle polarization for cadC; impulsively excited vibrations; Cartesian coordinates (QM region only) (PDF)
