# Supplementary material for: Unified Description of Ultrafast Excited State Decay Processes in Epigenetic Deoxycytidine Derivatives
Source: J Phys Chem Lett. 2021 Nov 8;12(45):11070–7. doi: 10.1021/acs.jpclett.1c02909 (PMC8607503; doi:10.1021/acs.jpclett.1c02909)
Supplement: Supplementary file 1 — jz1c02909_si_001.pdf [file jz1c02909_si_001.pdf]

Supporting Information:

Unified Description of Ultrafast Excited State Decay Processes in Epigenetic Deoxycytidine Derivatives

Piotr Kabacinski[†°], *Marco Romanelli*[‡°], *Eveliina Ponkkonen*[§], *Vishal Kumar Jaiswal*[‡], *Thomas Carell*[§], *Marco Garavelli*[‡*], *Giulio Cerullo*[†*], *Irene Conti*[‡*]

† IFN-CNR, Dipartimento di Fisica, Politecnico di Milano, Piazza Leonardo da Vinci 32, I-20133 Milano, Italy

‡ Dipartimento di Chimica Industriale, Università degli Studi di Bologna, Viale del Risorgimento 4, I-40136 Bologna, Italy

§ Department of Chemistry, Ludwig-Maximilians-Universität München, Butenandtstrasse 5-13, Munich 81377, Germany

° These authors contributed equally to the work

1. **Computational details**
2. **5-methyl-2'-deoxycytidine: critical points, conical intersections calculations.**
   2.1 Benchmark calculations
3. **5-hydroxymethyl-2'-deoxycytidine: time-resolved spectra, decay paths, critical points and conical intersections calculations.**
4. **5-formyl-2'-deoxycytidine: critical points and conical intersections calculations.**
   4.1 5-formyl-2'-deoxycytidine: formyl in *syn* and *anti* conformation
5. **5-carboxyl-2'-deoxycytidine: critical points and conical intersections calculations.**
6. **Chemical synthesis**
7. **Sample preparation**

8. **TA setup description**

9. **DUV probe measurement of fdC**

10. **Parallel polarizations for mdC and hmdC, and magic angle polarization for cadC**

11. **Impulsively excited vibrations**

12. **Measurement of pure solvent**

13. **Cartesian coordinates (QM region only)**

1. Computational details

1.1 Classical molecular dynamics

Each compound initial structure ,prior to QM/MM calculations, was obtained through classical molecular dynamics simulation, using the AMBER 12 suite[1]. Since no standard force-fields are available for these epigenetic derivatives, the utility *antechamber* of AMBER was used to generate semi-empirically AM1-BCC[2] charges and atom types from the AMBER GAFF force field[3]. Each molecule was surrounded by a cubic solvent box of TIP3P water molecules (force field: leaprc.water.tip3p) built up adding solvent molecules along x,y,z -directions within 30 Å from the nucleoside and periodic boundary conditions were applied. Before running final production runs of 100 ns, each system was initially optimized (2000 steps of optimization), then heated at constant volume from 0K to 300K for 20 ps and subsequently equilibrated at 300K for 300 ps at constant pressure (1atm), allowing the density of the system to relax. Hydrogen-containing bonds were restrained by using the SHAKE algorithm, electrostatic and non-bonding interactions were evaluated with a cutoff of 10 Å and the time-step was set to 2 fs.
Then, each production run of 100 ns has been analyzed by means of cluster analysis using the clustering algorithm *dbscan*[4], setting the RMSD of atomic positions as a distance metric (RMSD = 0.2 Å). For each compound, the molecular structure closest to the centroid of the most populated cluster along the whole dynamics has been chosen for further QM/MM calculations. By doing so It has also been possible to investigate the 'sugar-syn vs sugar-anti' conformational isomerism of these nucleosides around the N-glycosidic in water solution: all four systems show that the 'sugar-anti conformer' is predominant in water solutions at 300K and 1 atm, as suggested by previous works[5–7].

1.2 QM/MM setup

The QM/MM calculations were done by means of the COBRAMM[8] software, developed in our group, interfacing Molcas 8[9] and OpenMolcas[10,11] for the QM part and AMBER for the MM region. A spherical droplet centered on each nucleoside with a radius of 28 Å was cut out from the cubic water box used in the MM dynamics. The partitioning scheme employed was High/Medium/low (Figure S1), where the pyrimidine ring of each nucleoside was treated at QM-level (High layer part), the sugar ring plus water molecules at most 5 Å far from the high layer part were included in the medium layer (i.e. movable during QM/MM optimizations and coupled to the QM region), whereas the rest of the water droplet was included in the low layer (i.e. frozen during QM/MM optimizations).

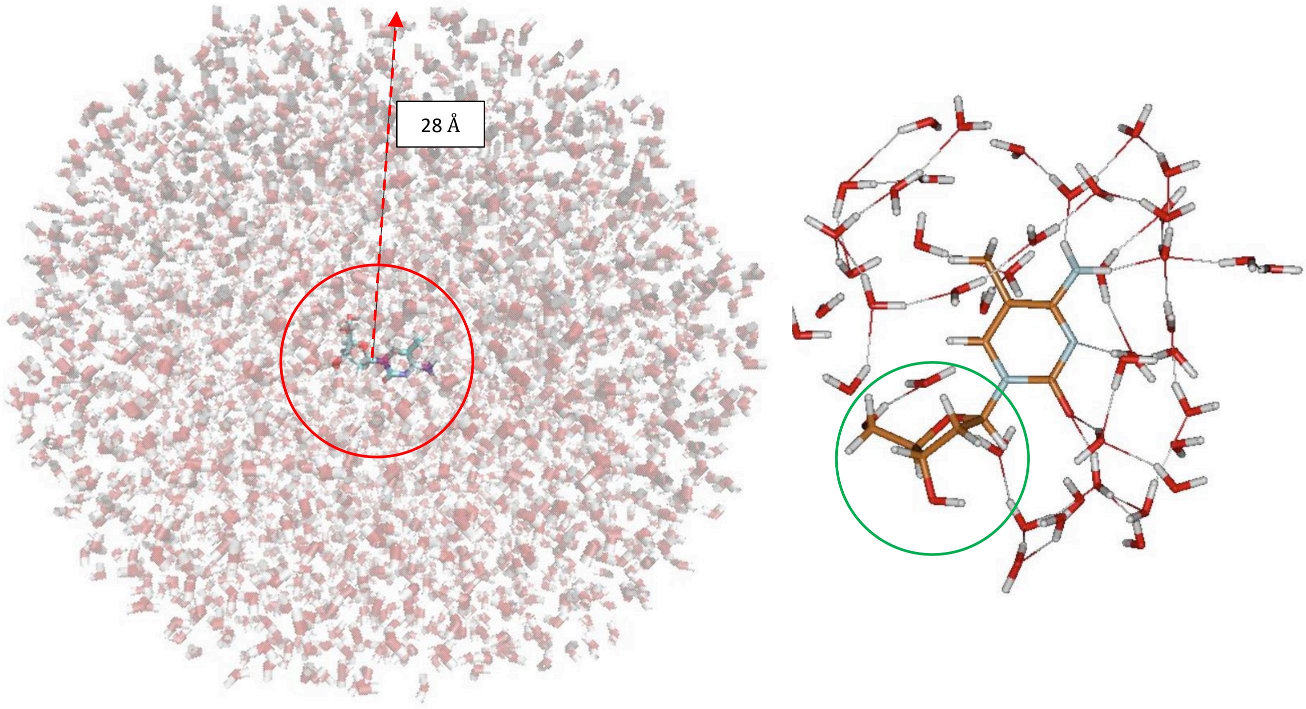

Figure S1: Spherical droplet with a radius of 28 Å used for the QM/MM calculations (left), High+Medium layers (right). The sugar moiety (green circle) is included in the movable Medium MM part.

1.3 QM/MM calculations

Ground state optimizations were done at the Møller–Plesset second-order perturbation theory (MP2) level as implemented in the Molcas8[9] package through its interface with COBRAMM by using the Gaussian16[12] optimizer. Excited states critical points and conical intersections (for all the four epigenetic nucleosides) have been obtained optimizing at QM(SS-CASPT2(14,10)/MM level, basis set ANO-L-VDZP (2s1p contraction on hydrogen atoms and 3s2p1d on carbon/oxygen/nitrogen atoms). The (14,10) active spaces used for all the four epigenetic bases optimizations is documented in Figure S2b, S3b, S4b and S5b, inside the grey squares, subgroups of the RAS-PT2 larger active space. The decay paths documented in Figure 2, 3, 4 and S7 (and in Figure S6, S8, S9 and S10) have been obtained calculating the vertical energies of the ground state minima and refining the vertical energies of each structure documented for all the decay paths at SS-RASPT2/ANO-L-VDZP level, using different active spaces and state-average values, depending on the nucleoside considered (documented in the captions of Figures S2, S3, S4, S5). SE signals have been calculated at the same SS-RASPT2 level, instead for the PA values a state-averaging on 30 states has been employed. The ionization-potential-electron-affinity (IPEA) shift[13] was set to 0.0 and an imaginary shift[14] of 0.2 a.u. was used. Conical intersection optimizations were performed with the gradient projection algorithm by Bearpark et al.[15] as implemented in COBRAMM.

a)

Excited state n.	Energy SS-RASPT2(4,7|0|4,7)-SA9 (eV/nm)	Transition character (Coefficient)	Oscillator strength (f)
$S_1(\pi\pi_1^*)$	4.54/273	H→L (0.7)	0.07
$S_2(\pi\pi_2^*)$	5.09/244	H-1→L (0.54)<br>n_N→L (0.14)	0.14
$S_3(n_N\pi^*)$	5.40/230	n_N→L (0.64)<br>H-1→L (0.11)	0.07
S_4	5.92/209	H→L+1 (0.41)<br>H-1→L+1 (0.21)	0.33
$S_5(n_O\pi^*)$	5.95/208	n_O→L (0.43)<br>n_O→L+1(0.26)	0.01
S_6	6.15/202	n_O→L+1(0.41)<br>n_O→L (0.3)	0.01
S_7	6.23/199	H→L+1 (0.28)<br>H-1→L+1 (0.24)<br>n_N→L+1(0.1)	0.60

b)

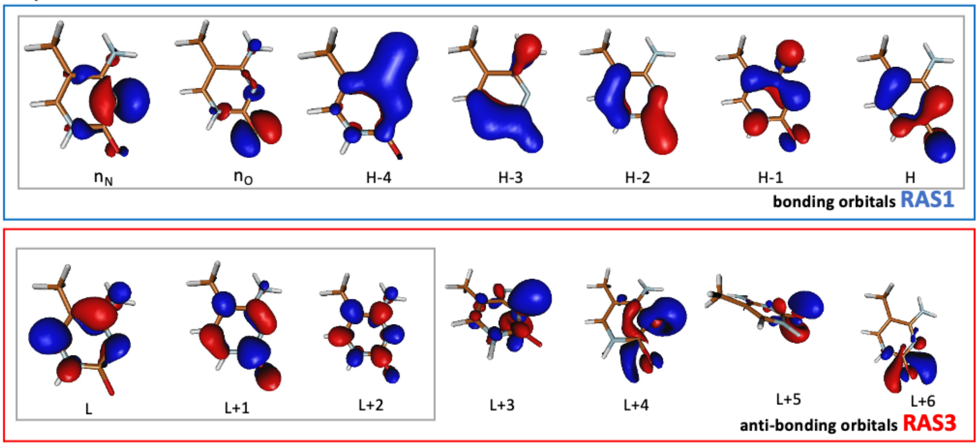

Figure S2: a) Vertical absorption energies of 5-methyl-2'-deoxycytidine computed at SS-RASPT2/SA-9 RASSCF(4,7|0|4,7)/ ANO-L-VDZP. b) QM(RASPT2)/MM active space; grey squares include the (14,10) active space adopted for all the excited state optimizations at QM(SS-CASPT2(14,10)/MM level.

a)

Excited state n.	Energy SS-RASPT2(4,7|0|4,7) (eV/nm)	Transition character (Coefficient)	Oscillator strength (f)
S_1 ($\pi\pi_1^*$)	4.56/272	H→L (0.67)	0.07
S_2 ($\pi\pi_2^*$)	5.08/244	H-1→L (0.64)	0.23
S_3 ($\pi\pi_3^*$)	5.77/215	H-1→L+1 (0.53) H→L+1 (0.07)	0.17
S_4 ($n_N\pi^*$)	5.88/211	n_N→L (0.8)	0.01
S_5 ($n_O\pi^*$)	6.08/204	n_O→L (0.52) n_O→L+1(0.25)	0
S_6	6.18/201	n_O→L+1(0.56) n_O→L (0.22)	0.01
S_7	6.22/199	H→L+1 (0.64) H-1→L (0.07)	0.88

b)

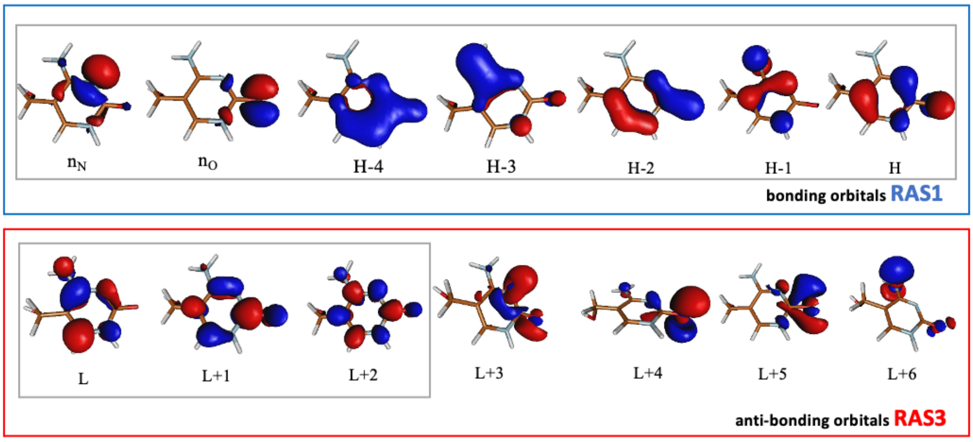

Figure S3: a) Vertical absorption energies of 5-hydroxymethyl-2'-deoxycytidine computed at SS-RASPT2/SA-8 RASSCF(4,7|0|4,7)/ ANO-L-VDZP). b) QM(RASPT2)/MM active space; grey squares include the (14,10) active space adopted for all the excited state optimizations at QM(SS-CASPT2(14,10)/MM level.

a)

Excited state n.	Energy SS-RASPT2(4,9|0|4,7) (eV/nm)	Transition character (Coefficient)	Oscillator strength (f)
S_1 ($\pi\pi_1$*)	4.36/284	H→L (0.5)<br>H→L+1 (0.08)	0.04
S_2 ($n_O\pi$*-f/$n_N\pi$*)	4.40/282	n_O-f/n_N→L (0.42)<br>n_N/n_O-f→L (0.14)	0
S_3 ($\pi\pi_2$*)	4.56/272	H-1→L (0.52)	0.03
S_4 ($\pi\pi_3$*)	5.09/244	H→L+1 (0.38)<br>n_O-c→L (0.12)<br>H→L (0.08)	0.57
S_5 ($n_O\pi$*-c)	5.76/215	n_O-c→L (0.47)<br>n_O-c→L+1 (0.10)	0.13
S_6	5.84/212	n_N/n_O-f→L (0.27)<br>n_O-f/n_N→L (0.13)<br>n_O-f/n_N→L+1 (0.12)	0.04
S_7	6.43/193	n_O-c→L+1 (0.53)<br>n_O-c→L (0.10)	0.03

b)

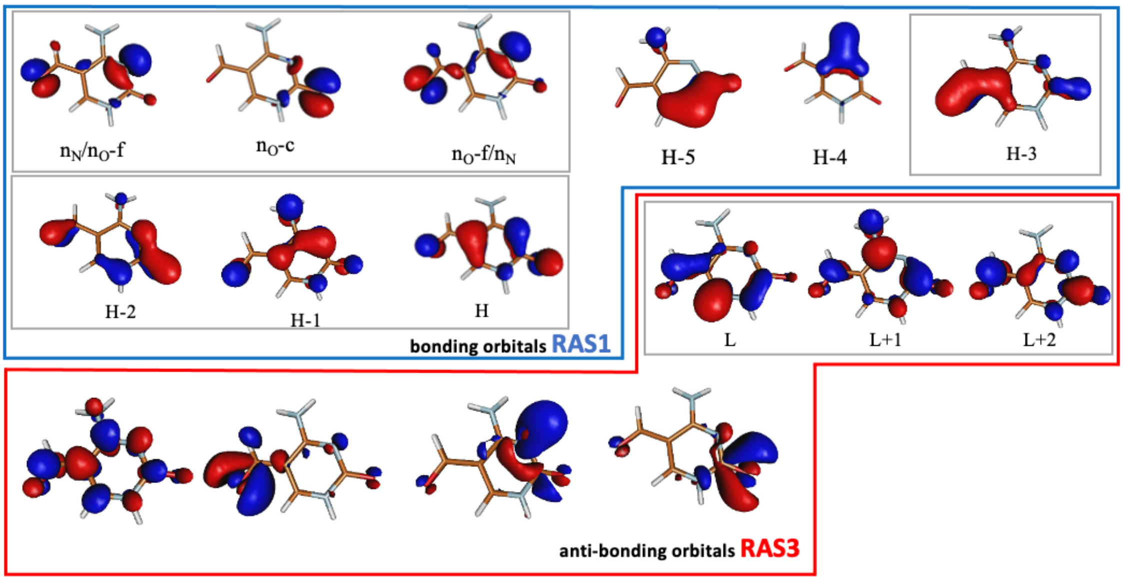

Figure S4: a) Vertical absorption energies of 5-formyl-2'-deoxycytidine computed at SS-RASPT2/SA-8 RASSCF(4,9|0|4,7)/ ANO-L-VDZP. The state S_2 is simply labeled in the main text as $n\pi^*$. Here in parentheses we specify that this np* state is localized on both the oxygen of the formyl group and on the nitrogen of the pyrimidine ring: see the corresponding orbital picture in panel b. b) QM(RASPT2)/MM active space; grey squares include the (14,10) active space adopted for all the excited state optimizations at QM(SS-CASPT2(14,10)/MM level.

a)

Excited state n°	Energy SS-RASPT2(4,10|0|4,7) (eV/nm)	Transition character (Coefficient)	Oscillator strength (f)
$S_1(\pi\pi_1^*)$	4.46/278	H→L (0.60)<br>H-1→L+1 (0.13)	0.04
S_2 $(\pi\pi_2^*)$	4.96/250	H-1→L (0.63)	0.12
S_3 $(\pi\pi_3^*)$	5.19/239	H→L+1 (0.26)<br>H-1→L+1 (0.32)	0.23
S_4 $(n_O\pi^*$-ca)	5.19/239	n_O-ca→L+1 (0.35)<br>n_O-ca→L (0.21)	0
S_5 $(\pi\pi_4^*)$	5.77/215	H→L+1 (0.40)<br>H-1→L+1 (0.19)	0.86
S_6 $(n_N\pi^*/n_O\pi^*$-c)	5.98/207	n_N/n_O-c→L (0.37)<br>n_O-c/ n_N→L (0.27)	0.01
S_7 $(n_O\pi^*$-c/ $n_N\pi^*)$	6.41/194	n_O-c/ n_N→L+1(0.28)<br>n_O-c/ n_N→L+1(0.17)<br>n_N/n_O-c→L+1 (0.15)<br>n_N/n_O-c→L (0.11)	0.01

b)

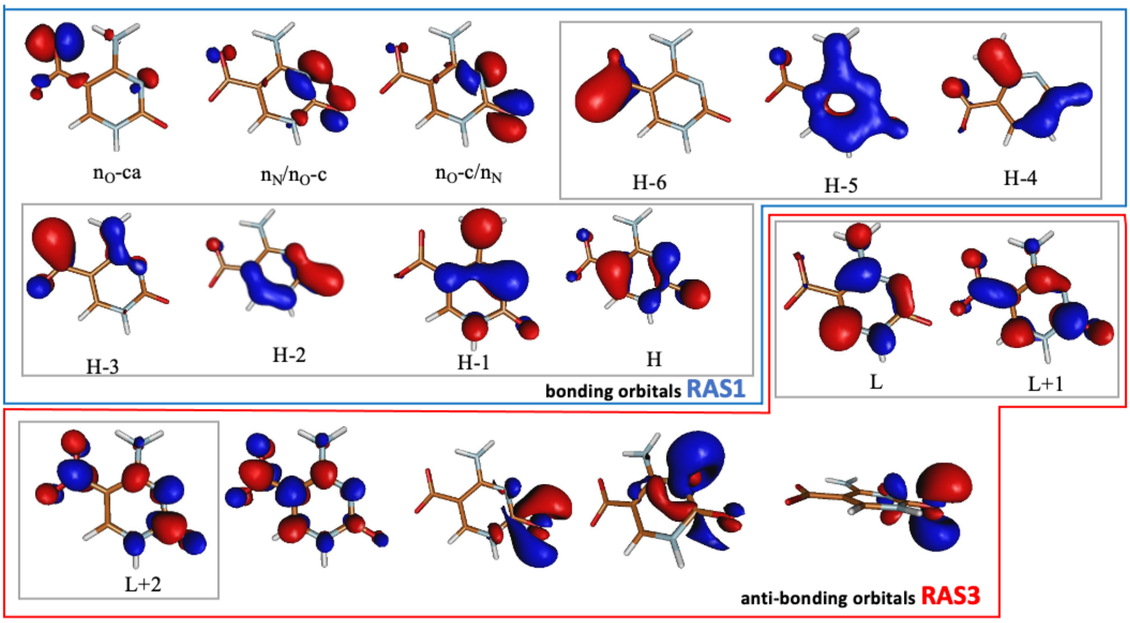

Figure S5: a) Vertical absorption energies of 5-carboxyl-2'-deoxycytidine computed at SS-RASPT2/SA-8 RASSCF(4,10|0|4,7)/ ANO-L-VDZP. . b) QM(RASPT2)/MM active space; grey squares include the (14,10) active space adopted for all the excited state optimizations at QM(SS-CASPT2(14,10)/MM level.

2. 5-methyl-2’-deoxycytidine: critical points, conical intersections calculations.

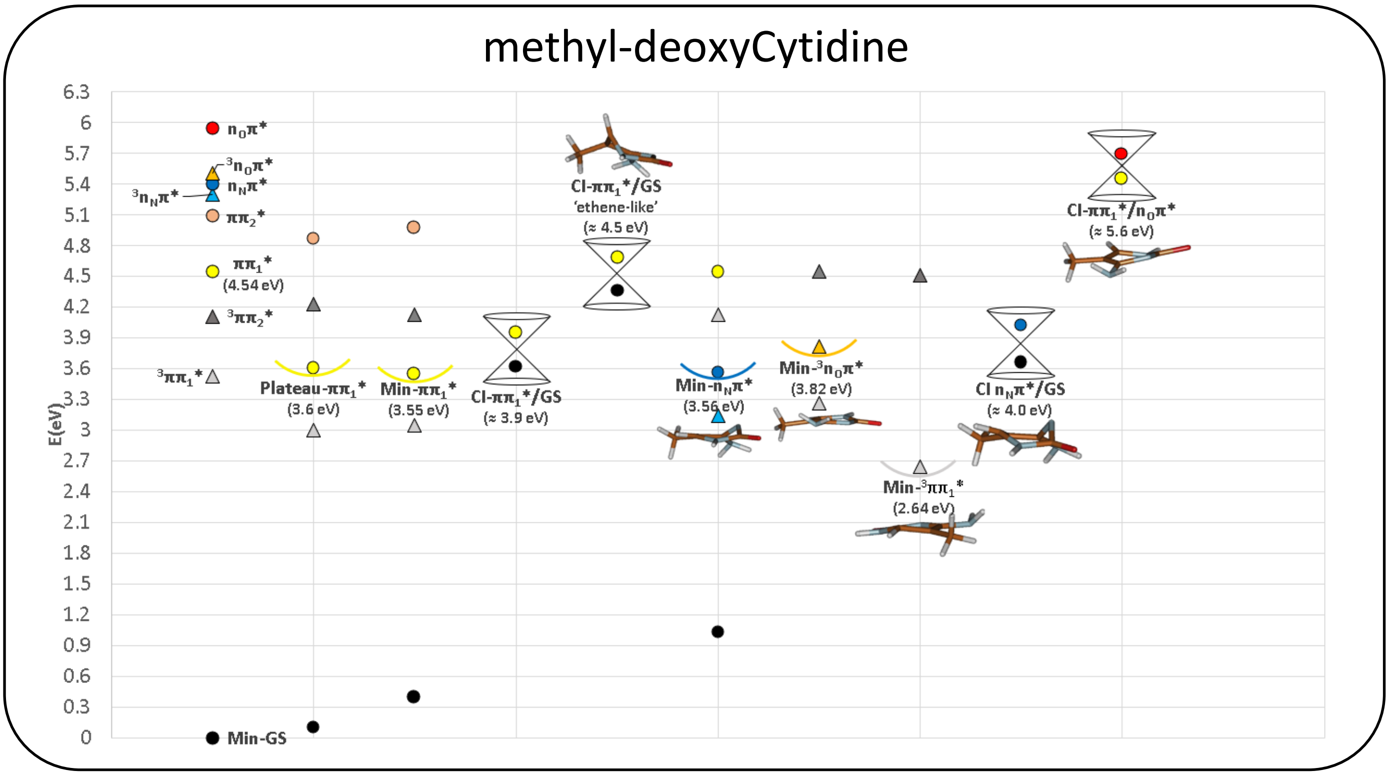

Figure S6: All the critical points and conical intersections of 5-methyl-2’-deoxycytidine optimized at QM(SS CASPT2(14,10)/MM level. The energy values reported in eV are computed at SS-RASPT2/SA-9 RASSCF(4,7|0|4,7)/ ANO-L-VDZP and they are relative to the ground state minimum energy value (Min S_0). On top of each optimized minimum (colored semicircles), there are reported low-lying singlet (colored circles) and triplet (colored triangles) excited states.

The CI-$\pi\pi_1$*/GS of the 5-methyl-2’-deoxycytidine characterized in the present work is not exactly reproducing the structure of the ‘ethene-like’ CI reported previously by Martinéz-Fernandéz et al. [16] but it is exactly on the reaction coordinate distortion that leads from the FC region to the Plateau-$\pi\pi_1$*, so right along the distortion mode dynamically populated (See on top of Figure 2d). It features a ring puckering (as the ethene-like CI) but characterized by the NH_2 group out of plane motion as a consequence of the torsion around the C_4-C_5 bond, instead of the C_5-C_6 involved in the ethene-like CI. The difference with the CI structure found by Martinéz-Fernandéz et al. could be assigned to some variance in the calculation level (i.e. different QM/MM setup including smaller solvent sphere) and more probably to the system configuration: they analyzed the sugar-syn instead of the most populated sugar-anti conformer we adopted, which could hinder some particular ring distortion. In fact, we also characterized the ‘ethene-like’ CI, but we found that it lies at higher energy (≈0.50 eV above our documented CI, Figure S6).

2.1 Benchmark calculations

Benchmarking the method employed is mandatory to validate its accuracy and reliability, in particular when investigating different molecular systems. Indeed, before starting extensive computational investigations, we performed systematic tests and benchmarks. In particular, the following was tested and verified:

i) reliability and accuracy of the QM-MM partitioning.
ii) the flavor of multireference perturbative approach systematically employed in the work;
iii) the active space.

the choice of the active space has been carefully analyzed to find a suitable methodology able to provide the best agreement with the experimental linear absorption spectrum, focusing on the lowest $S_0 \rightarrow S_1$ transition, which is exactly the main band that we largely populate adopting a 4.35 eV (285 nm) pump energy, as documented in ref. [17]. 5-methyl-dC (5mdC) is the first nucleoside that we started investigating, and thus we considered it as a test-case for such purpose. Tables S1, S2 and S3 report our tests on 5mdC vertical excitation energies that were calculated employing different active spaces, different levels of theory (SS vs MS, or CASPT2 vs RASPT2) and different QM/MM partitioning (all details are specified in each first row of the tables). Our choice to adopt the SS-RASPT2 method (on top of ground state MP2-optimized geometries) is based on the documented results, trying to reach the best agreement with the lowest experimental absorption bands in the UV.

The final choice was the MP2 GS minimum, to coherently use a second order perturbative methods on both the ground and CASPT2 excited states calculations. Multireference methods have been adopted because they properly describe the internal conversion and inter-system crossing regions.

Transition characters in Tables S1-S3 are simply associated to $\pi\pi^*$ or $n\pi^*$ transitions (or mixed). Differently, Figures from S2 to S5 shows proper states labels, addressable to the corresponding orbitals exhibited in the same picture.

<table>
<tr><td colspan="7">Ground state MP2 optimized structure
(Sugar ring in the MM part , nucleobase ring in the QM one)</td></tr>
<tr><td>State n.</td><td>MS-CASPT2 (14,10) in eV</td><td>Transition character</td><td>Osc. strength</td><td>SS-CASPT2 (14,10) in eV</td><td>Transition character</td><td>Osc. strength</td></tr>
<tr><td>1</td><td>4.25</td><td>π- π*</td><td>0.33</td><td>4.27</td><td>π-π*</td><td>0.07</td></tr>
<tr><td>2</td><td>4.64</td><td>π-π*</td><td>0.08</td><td>4.98</td><td>n/π-π*</td><td>0.08</td></tr>
<tr><td>3</td><td>5.46</td><td>n-π*</td><td>0.06</td><td>4.99</td><td>n/π-π*</td><td>0.08</td></tr>
<tr><td>4</td><td>5.5</td><td>n/π-π*</td><td>0.2</td><td>5.32</td><td>n-π*</td><td>0.01</td></tr>
<tr><td>State n.</td><td>MS-RASPT2 (4,7|0|4,7) in eV</td><td>Transition character</td><td>Osc. strength</td><td>SS-RASPT2 (4,7|0|4,7) in eV</td><td>Transition character</td><td>Osc. strength</td></tr>
<tr><td>1</td><td>4.54</td><td>π-π*</td><td>0.16</td><td>4.54</td><td>π-π*</td><td>0.07</td></tr>
<tr><td>2</td><td>4.97</td><td>π-π*</td><td>0.26</td><td>5.09</td><td>n/π-π*</td><td>0.14</td></tr>
<tr><td>3</td><td>5.57</td><td>n-π*</td><td>0.01</td><td>5.4</td><td>n/π-π*</td><td>0.07</td></tr>
<tr><td>4</td><td>5.87</td><td>n/π-π*</td><td>0.15</td><td>5.92</td><td>π-π*</td><td>0.33</td></tr>
<tr><td colspan="7">Experimental absorption maxima</td></tr>
<tr><td colspan="7">4.46 eV</td></tr>
<tr><td colspan="7">5.17 eV</td></tr>
<tr><td colspan="7">5.79 eV</td></tr>
</table>

Table S1: Vertical excitation energy of 5-methyl-2'-deoxycytidine computed at different levels of theory starting from a ground state optimized structure where the QM part (only nucleobase here) was treated at MP2 level. The sugar ring is in the MM layer. The active spaces refer to Figure S2 above.

Ground state DFT/PBE0 optimized structure (basis set for optimization def2tvzp) (Full nucleoside optimized at QM level, but VEEs below were obtained by including the sugar ring in the MM part and the remaining nucleobase ring in the QM one)						
State n.	MS-CASPT2 (14,10) in eV	Transition character	Osc. strength	SS-CASPT2 (14,10) in eV	Transition character	Osc. strength
1	4.37	π-π*	0.26	4.35	π-π*	0.07
2	4.88	π-π*	0.2	4.99	n/π-π*	0.14
3	5.63	n-π*	0	5.43	n-π*	0.02
4	5.77	π-π*	0.24	5.72	n-π*	0.03
State n.	MS-RASPT2 (4,7|0|4,7) in eV	Transition character	Osc. strength	SS-RASPT2 (4,7|0|4,7) in eV	Transition character	Osc. strength
1	4.67	π-π*	0.19	4.69	π-π*	0.07
2	5.17	π-π*	0.26	5.25	n/π-π*	0.15
3	5.77	n-π*	0.01	5.63	n/π-π*	0.08
4	5.98	π-π*	0.21	5.99	π-π*	0.27
Experimental absorption maxima						
4.46 eV						
5.17 eV						
5.79 eV						

Table S2: Vertical excitation energy of 5-methyl-2'-deoxycytidine computed at different levels of theory starting from a ground state optimized structure where the QM part (full nucleoside here) was treated at DFT/PBE0 level. In the VEEs instead, the sugar ring was treated at MM level. The active spaces refer to Figure S2 above.

Ground state DFT/PBE0 optimized structure (basis set for optimization def2tvzp) (Full nucleoside at QM level, also for VEEs below)						
State n.	MS-CASPT2 (14,10) in eV	Transition character	Osc. strength	SS-CASPT2 (14,10) in eV	Transition character	Osc. strength
1	4.32	π-π*	0.3	4.34	π-π*	0.07
2	4.84	π-π*	0.18	4.94	n/π-π*	0.14
3	5.6	n-π*	0.01	5.35	n/π-π*	0.04
4	5.64	π-π*	0.2	5.61	n-π*	0.04
State n.	MS-RASPT2 (4,7|0|4,7) in eV	Transition character	Osc. strength	SS-RASPT2 (4,7|0|4,7) in eV	Transition character	Osc. strength
1	4.53	π-π*	0.23	4.57	π-π*	0.08
2	5.13	π-π*	0.23	5.18	n/π-π*	0.17
3	5.76	n-π*	0.02	5.63	n/π-π*	0.07
4	5.87	π-π*	0.2	5.96	n/π-π*	0.2
Experimental absorption maxima						
4.46 eV						
5.17 eV						
5.79 eV						

Table S3: Vertical excitation energy of 5-methyl-2'-deoxycytidine computed at different levels of theory starting from a ground state optimized structure where the QM part (full nucleoside here) was treated at DFT/PBE0 level. Also the VEEs were obtained by including the whole compound in the QM region. The active spaces refer to Figure S2 above.

3. **5-hydroxymethyl-2'-deoxycytidine: time-resolved spectra, decay paths, critical points and conical intersections calculations.**

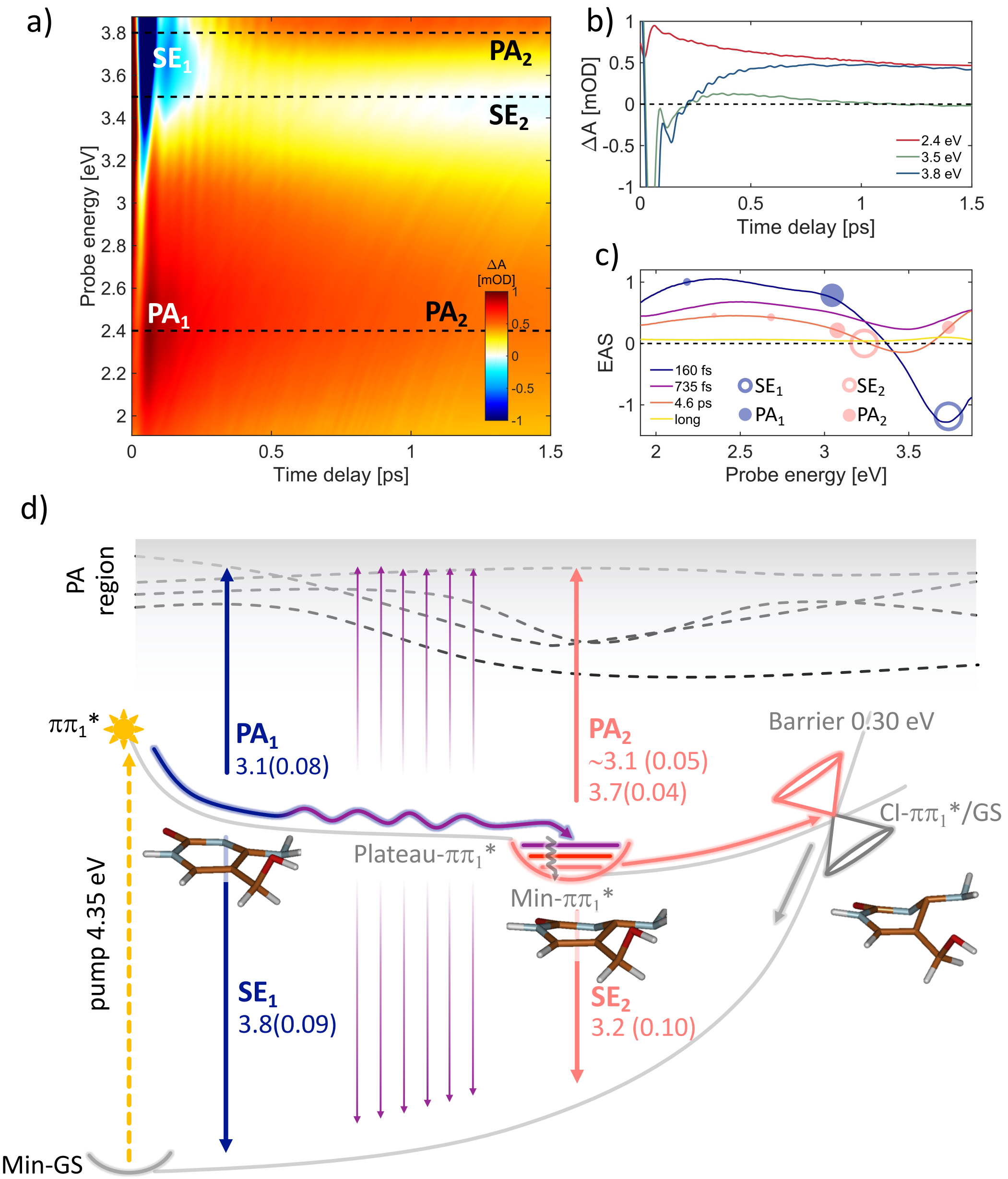

Figure S7: (a) ΔA map of hmdC in water solution recorded with pulse polarizations set at magic angle, (b) dynamics at selected probe energies (eV), (c) EAS with the corresponding time constants: 160 fs (blue curve) is the time needed to relax from the FC to the Plateau-$\pi\pi_1$* flat region (see calculated paths in d panel), 735 fs (purple curve) corresponds to the time lived in the Plateau-$\pi\pi_1$* region to fully relax in the Min-$\pi\pi_1$*(wavy purple line, panel d). The 4.6 ps time constant (pink curve panel c) relates to the $\pi\pi_1$*-GS decay process (CI-$\pi\pi_1$*/GS, panel d), involving a 0.350eV barrier. Yellow line corresponds to long-lived products probably due to other minor decay paths. Empty and full circles correspond to the calculated SE and PA energy values, respectively, and the circles dimensions are proportional to the computed oscillator strength values (documented in panel d). (d) The colored curves illustrate the hypothesized decay paths of hmdC, optimized at QM(SS CASPT2(14,10)/MM level, along the reaction co-ordinate leading to the CI-$\pi\pi_1$*/GS. Photoinduced absorption (PA) were computed at SS-RASPT2/SA-30 RASSCF(4,7|0|4,7)/ ANO-L-VDZP while SA-8 was employed for the stimulated emission signals (SE). Critical points energies in Figure S8. Oscillator strengths are reported in brackets. Decay paths and SE/PA colors arrows are matching with the lines colors of time constants in panel c (EAS). Molecular structures refer to QM region only.

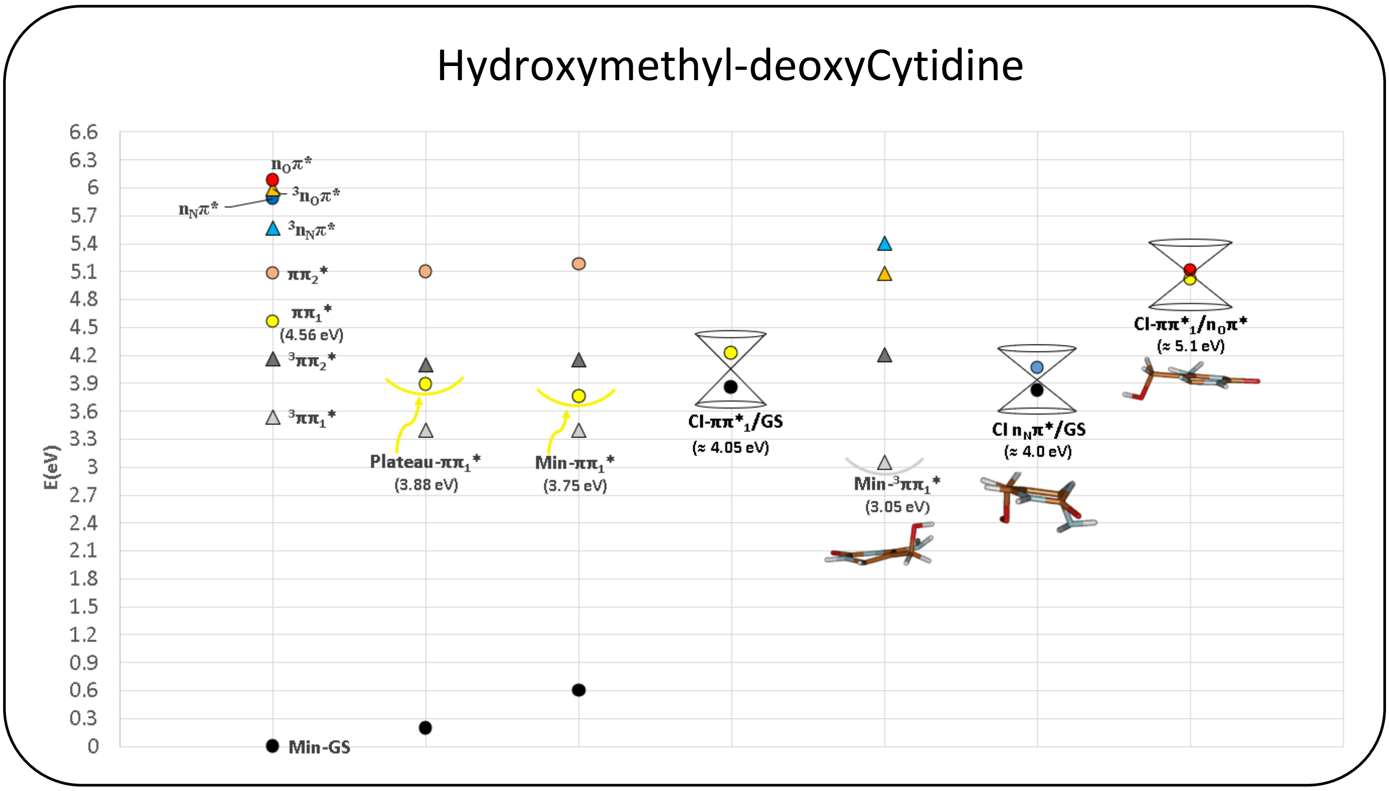

Figure S8: All the critical points and conical intersections of 5-hydroxymethyl-2'-deoxycytidine optimized at QM(SS CASPT2(14,10)/MM level. The energy values reported in eV are computed at SS-RASPT2/SA-8 RASSCF(4,7|0|4,7)/ ANO-L-VDZP and they are relative to the ground state minimum energy value (Min S_0). On top of each optimized minimum (colored semicircles), there are reported low-lying singlet (colored circles) and triplet (colored triangles) excited states.

4. 5-formyl-2’-deoxycytidine: critical points and conical intersections calculations.

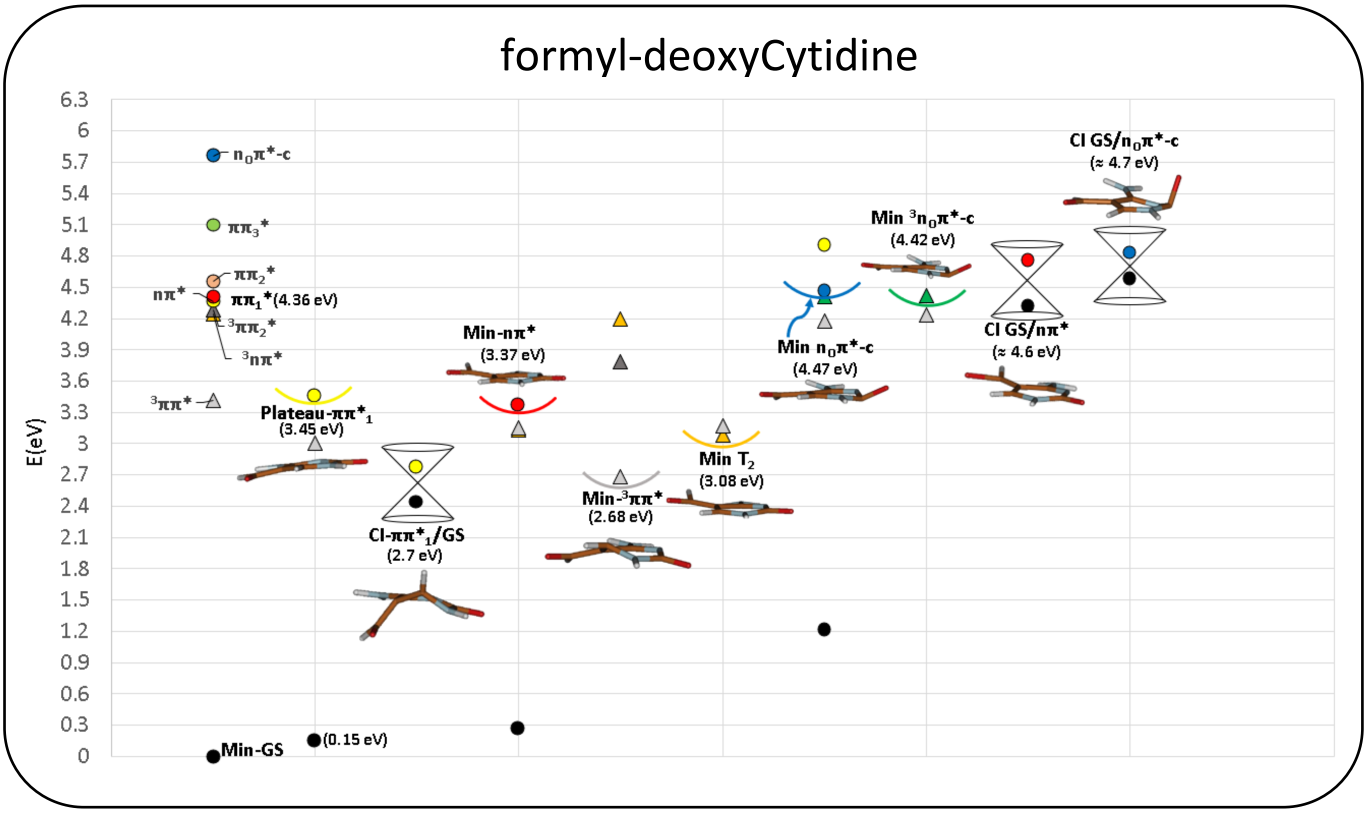

Figure S9: All the critical points and conical intersections of 5-formyl-2’-deoxycytidine optimized at QM(SS CASPT2(14,10)/MM level. The energy values reported in eV are computed at SS-RASPT2/SA-8 RASSCF(4,9|0|4,7)/ ANO-L-VDZP and they are relative to the ground state minimum energy value (Min S_0). On top of each optimized minimum (colored semicircles), there are reported low-lying singlet (colored circles) and triplet (colored triangles) excited states.

4.1 5-formyl-2’-deoxycytidine: formyl in *syn* and *anti* conformation

The fdC conformer not exhibiting any intramolecular hydrogen bond between the amino and the formyl groups (named *anti conformer*) was mainly discussed in this study, not showing the one where formyl carbonyl and the amino group are bridged through an intramolecular H-bonding (named *syn conformer*). Despite the fact that both conformers may exist in a polar protic solvent (water), as discussed in a recent work[18], this choice was taken because the *anti conformer* is the one that, we believe, is more relevant for the ultrafast sub-400 fs photoinduced dynamics observed and investigated here. This choice was based on the following reasons:

i) While the *syn conformer* is expected to prevail in apolar solvents, the stability of the *anti conformers* smoothly increases while increasing the solvent polarity;

ii) In a polar protic solvent like water, that is able to give H-bonds stronger than the amino group itself, the stability of the two conformers should be similar if not event inverted. Moreover, the stronger molecular rigidity induced by an intramolecular H-bond should increase the entropic component of the system, making this conformer even less stable than the *anti* one where this restriction does not apply.

iii) More importantly, the O–H···:O H-bond strength (around 21 kJ/mol or 5.0 kcal/mol) is stronger than the N–H···:O (8 kJ/mol or 1.9 kcal/mol), further stabilizing the *anti conformer* adopted in our calculations.

iv) Additional Tests made on the fdC *syn conformer* show that during QM/MM $\pi\pi_1$* geometry optimization the initial intra-molecular N–H···:O bond, characterizing the FC region, is soon lost still along a barrierless path and it is replaced by the stronger O–H···:O H-bond with nearby solvent waters. This suggests that no hindrance in ring-puckering and/or formyl out of plane motions should appear afterwards, eventually pointing to very similar photoinduced dynamics for both conformers.

v) Finally, as a side note, neither the nπ* decay should be influenced by an intramolecular H-bond, because of the planar (not distorted) structure of the populated Min-nπ*, that resides in the ISC-nπ*/$^3\pi\pi$* region.

That said, it is apparent that the shortest (sub-400 fs) lifetimes experimentally observed should be properly assigned to the barrierless $\pi\pi_1$* decay path documented here for the *anti conformer*, as this does not suffer for any effect hindering the $\pi\pi_1$*→ GS internal conversion and the underlying out of plane motions as, instead, it may possibly occur in *syn conformers* if their intra-molecular N–H···:O bond persists also in the $\pi\pi_1$* excited state decay process. However, we have seen that this is unlike to occur (see point iv above). The striking agreement between computations and experiments support this view.

5. 5-carboxyl-2'-deoxycytidine: critical points and conical intersections calculations.

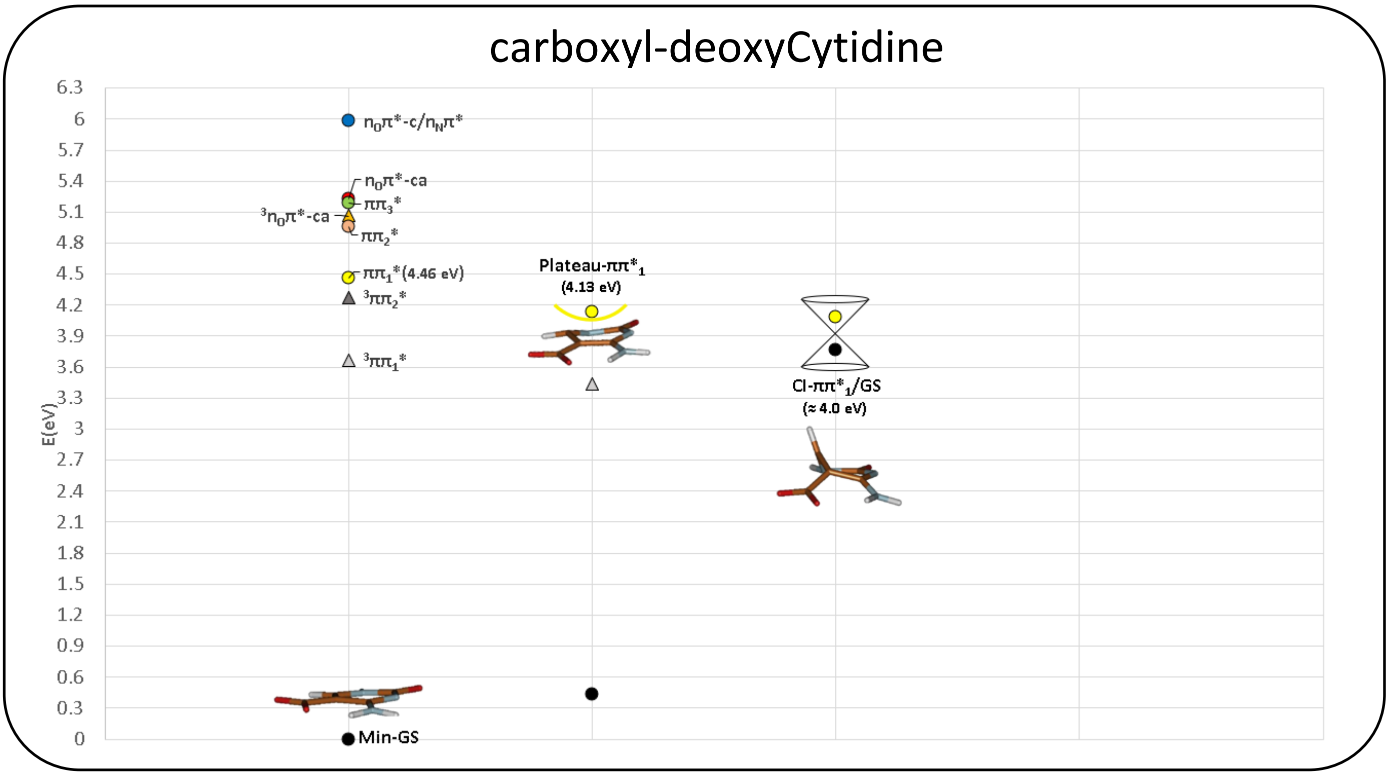

Figure S10: All the critical points and conical intersections of 5-carboxyl-2'-deoxycytidine optimized at QM(SS-CASPT2(14,10)/MM level. The energy values reported in eV are computed at SS-RASPT2/SA-8 RASSCF(4,10|0|4,7)/ ANO-L-VDZP and they are relative to the ground state minimum energy value (Min S_0). On top of each optimized minimum (colored semicircles), there are reported low-lying singlet (colored circles) and triplet (colored triangles) excited states.

6. Chemical synthesis

All the reactions (unless done in aqueous solutions) were performed using flame-dried glassware and under argon atmosphere. Chemicals were purchased from Sigma-Aldrich, TCI, Carbolution and Carbosynth. 5-methyl-2’-deoxycytidine was commercially available (Sigma-Aldrich). 5-hydroxymethyl-2’-deoxycytidine[19], 5-formyl-2‘-deoxycytidine[19] and 5-carboxyl-2’-deoxycytidine[20] were synthesized and purified according to previously published literature. Compounds were identified by NMR, High resolution MS and analytical HPLC.

7. Sample preparation for linear absorption/TA

A phosphate-buffered saline (PBS) has been prepared by dissolving 26.7 g of sodium dihydrogen phosphate and 20 g of sodium hydrogen phosphate in 1 L of ultrapure water to obtain a concentration of 150 mM and a pH of 7.1 after adjusting with sodium hydroxide. The sample solutions were prepared to achieve high concentrations for the TA measurement to preserve the high resolution of the experimental setup, for 2 OD in 100 µm: 5.1 mg of mdC in 1 mL, 5.8 mg of hmdC in 1 ml, 4.8 mg of fdC in 1 ml and 5.4 mg of cadC in 1 ml for 1.2 OD in 100 µm. For recording the steady-state absorption spectra the samples have been diluted 10 times to not saturate the spectrophotometer after going through the 1 mm path of the cuvette.

8. Setup description

Ultrafast TA experiments were performed using a home-built pump-probe setup[21], based on a Ti:Sapphire laser (Libra, Coherent) generating 100-fs pulses at 1.55 eV photon energy and 1 kHz repetition rate. A fraction of the laser power was used to feed a broadband visible non-collinear optical parametric amplifier (NOPA). The NOPA output pulses, with spectrum spanning 1.77-2.38 eV, were compressed to sub-10-fs duration by chirped dielectric mirrors and successively frequency doubled in a 20-µm-thick Type I β-barium borate crystal, generating broadband UV pump pulses with spectrum spanning 4.43-4.6 eV. The UV pulses were compressed with a MgF_2 prism pair to nearly transform-limited 18-fs duration, fully characterized by two-dimensional spectral interferometry[22]. Broadband probe pulses, covering 1.9-3.9 and 3.5-4.6 eV, were obtained through white light continuum generation by focusing either the laser fundamental wavelength or its second harmonic in a slowly moving 2-mm-thick CaF_2 plate. The instrumental response function of the system, depending on the probe wavelength, is estimated to be 25-35 fs.

To avoid photodamage of the sample and generation of solvated electrons by two-photon absorption from water, the pump energy was limited to 70 nJ (resulting in a fluence of 300 $\mu J/cm^2$) and high concentration sample solutions (~0.1 mm effective path length) were employed. TA spectra of the pure solvent, shown in Fig. S18, demonstrate the absence of solvated electrons. After the sample, the transmitted probe was sent to a spectrometer (SP2150 Acton, Princeton Instruments) and detected using a linear image sensor driven by a custom-built electronic board (Stresing Entwicklungsburo GmbH). For each probe wavelength, the differential absorption (ΔA) was measured as a function of the pump-probe delay.

9. DUV probe measurement of fdC

Figure S11 below shows the TA map of fdC probed in the DUV range to better highlight the strong PA signal at 4 eV discussed in the main text. In addition to this PA signal, we also observe the edge of the ground state bleach visible at the early times.

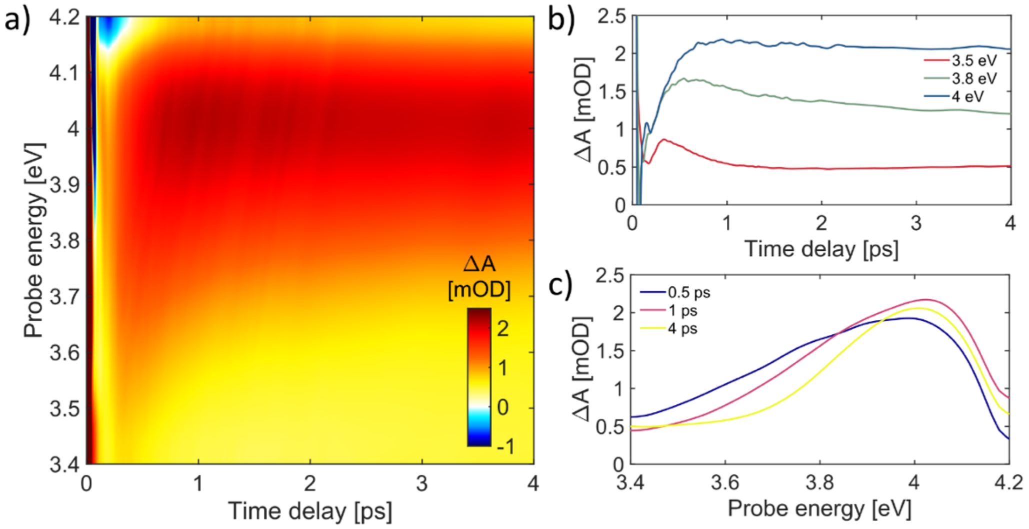

Figure S11: Transient absorption of fdC in DUV range: (a) TA map, (b) dynamics at selected energies, and (c) spectra at selected delays.

10. Parallel polarizations for mdC and hmdC and magic angle polarization for cadC

To complement the results shown and discussed in the main text, here we also report results obtained for different polarization settings. Those with parallel polarizations show relatively stronger SE signals, and the magic angle measurement for cadC shows better the PA signals hidden under the SE in the parallel version (shown in the main text). We did not observe a significant difference between magic angle and parallel measurement for fdC.

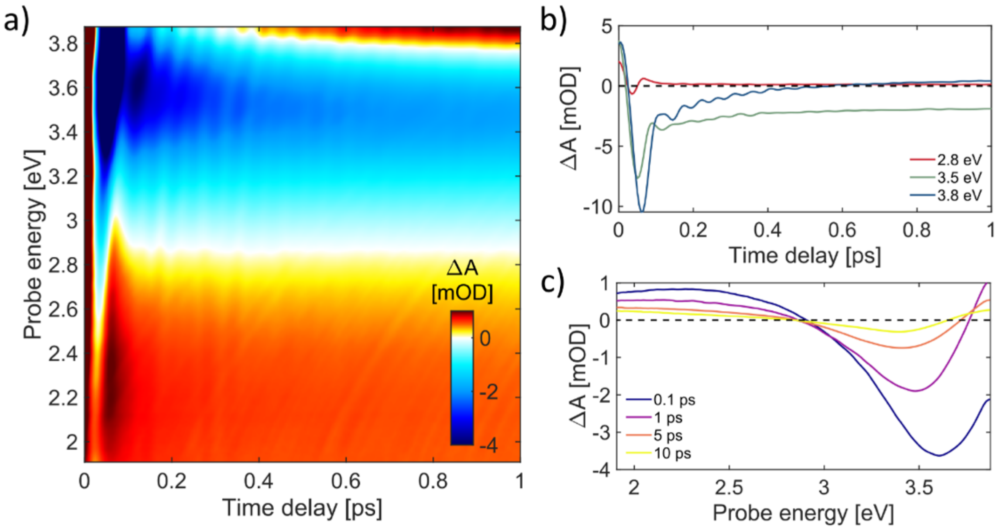

Figure S12: Transient absorption of mdC recorded with parallel polarizations: (a) TA map, (b) dynamics at selected energies, and (c) spectra at selected delays.

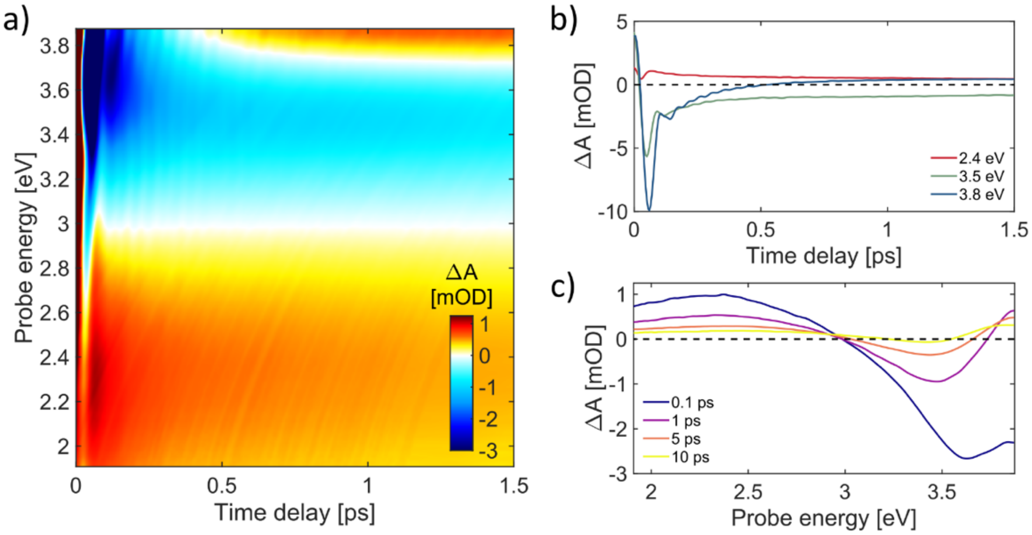

Figure S13: Transient absorption of hmdC recorded with parallel polarizations: (a) TA map, (b) dynamics at selected energies, and (c) spectra at selected delays.

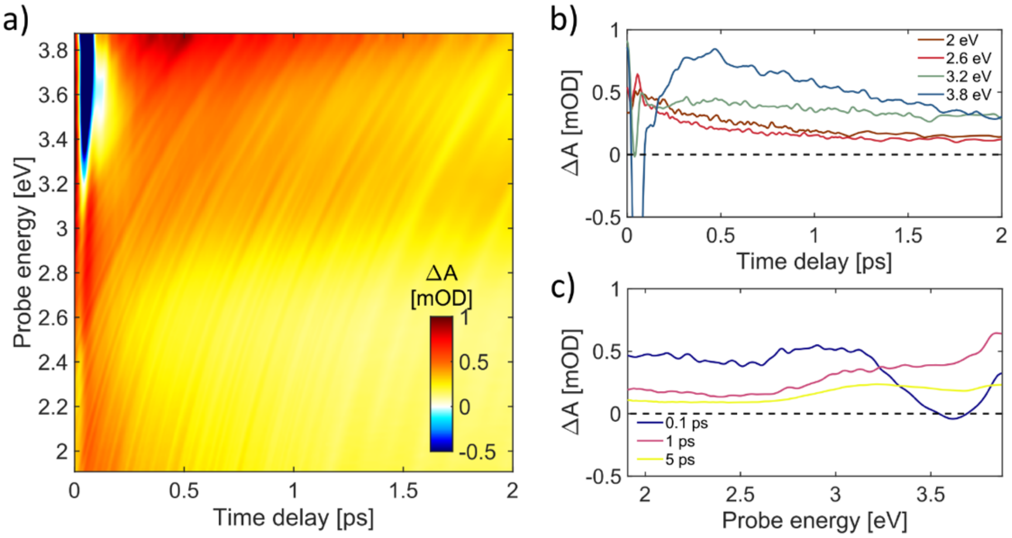

Figure S14: Transient absorption of cadC recorded with magic angle polarizations: (a) TA map, (b) dynamics at selected energies, and (c) spectra at selected delays.

11. Impulsively excited vibrations

Due to the high temporal resolution of our TA setup, we were able to observe fingerprints of coherent vibrations in the measured spectra. Below we show extracted oscillation maps together with their Fourier transforms. In the case of mdC and fdC, these are superimposed with the spectral density plots of the excited-state after vertical excitation from Franck-Condon region. The spectral density plots are computed with numerical CASPT2 frequencies, evaluated on the involved critical geometries of the populated excited-states.

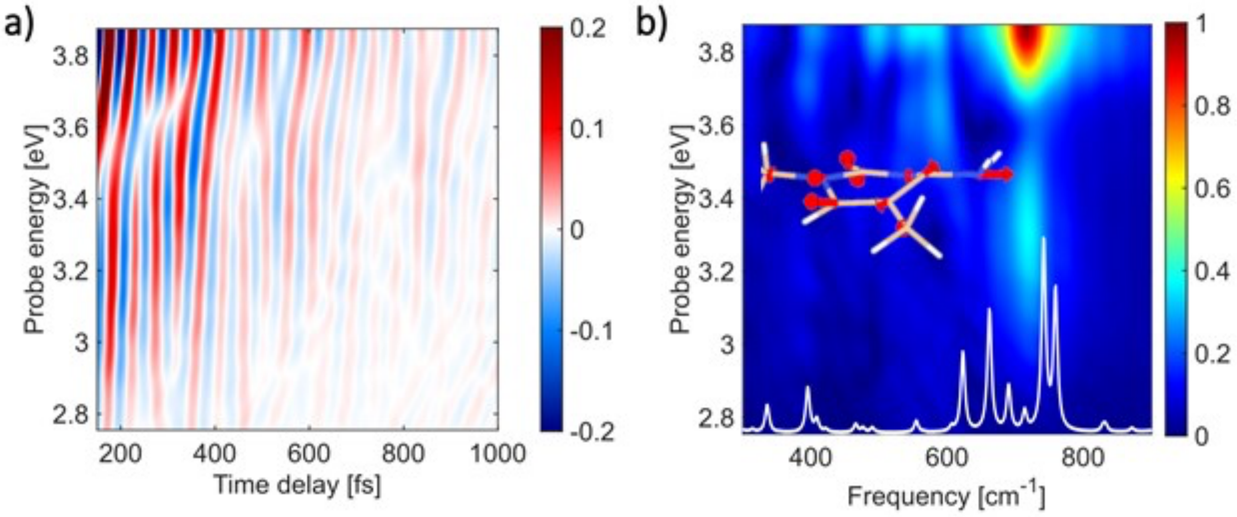

Figure S15: Oscillating residual of the transient absorption map for mdC (a), and its Fourier transform (b). We observe the dominant mode at 715 cm^{-1} with a node at 3.6 eV characteristic of excited state vibrations present on top of SE signal. The spectral density of the CASPT2 calculated normal modes of the Min-$\pi\pi_1$* is depicted with white lines in the lower part of panel (b), locating the most intense vibrations at 740 cm^{-1} (addressable to the experimental at 715 cm^{-1}) corresponding to the $\pi\pi_1$* ring distortion normal mode displayed in the figure.

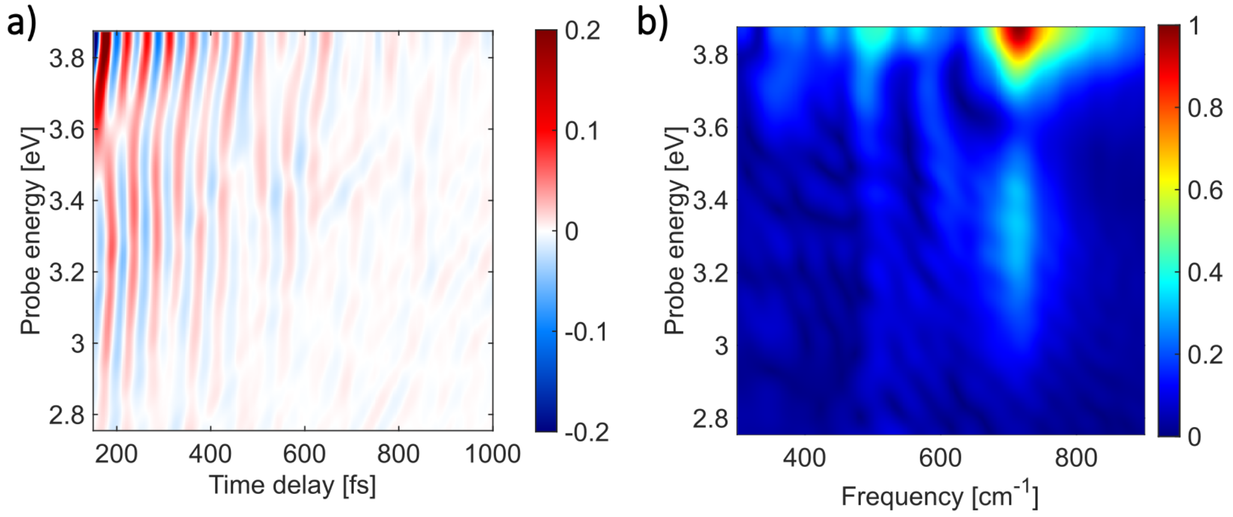

Figure S16: Oscillating residual of the transient absorption map for hmdC (a), and its Fourier transform (b). We observe the dominant mode at 715 cm^{-1} with a node at 3.6 eV characteristic of excited state vibrations present on top of SE signal.

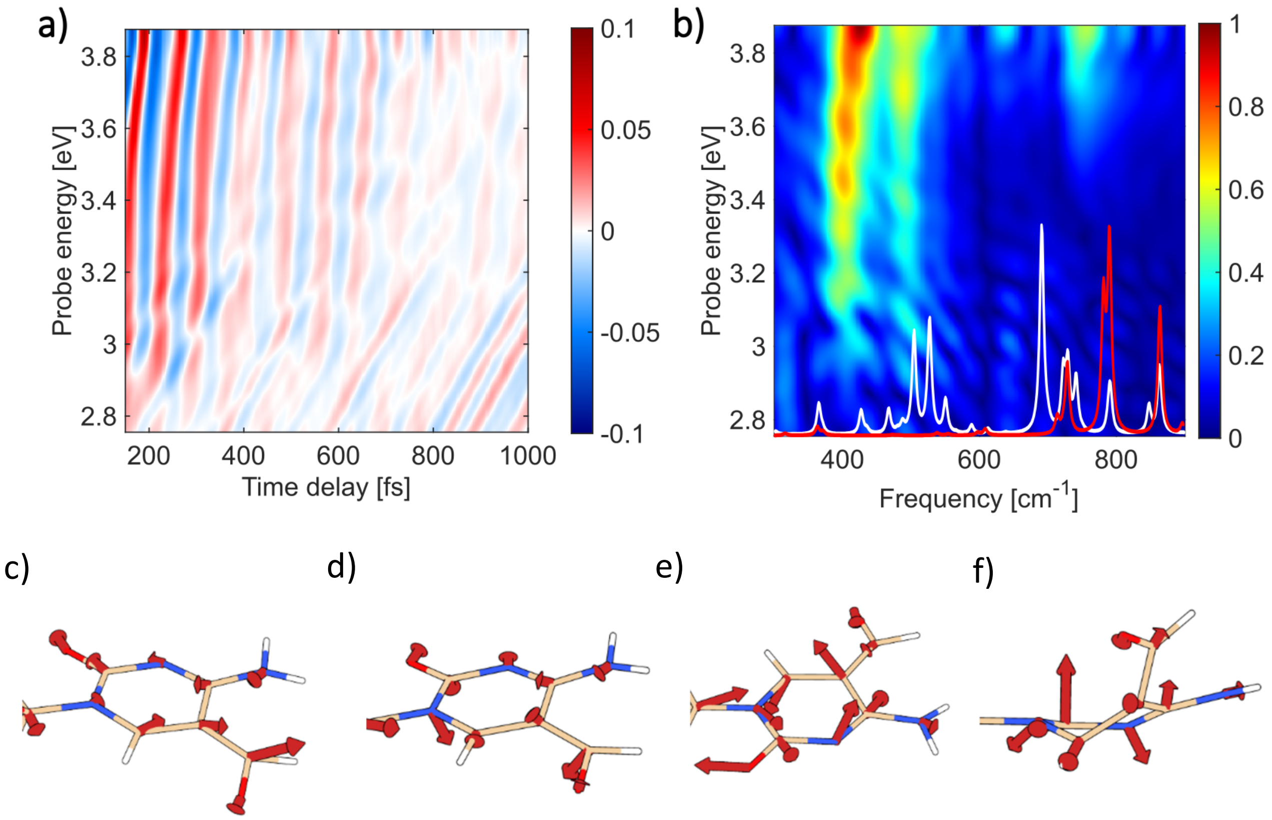

Figure S17: Oscillating residual of the transient absorption map for fdC (a), and its Fourier transform (b). We observe the dominant modes at 410 cm^{-1}, 490 cm^{-1} and 750 cm^{-1}. The CASPT2 calculated spectral density of the Plateau-$\pi\pi_1$* (red lines) locate the most intense vibrations at 791 cm^{-1} (close to the experimental at 750 cm^{-1}) corresponding to the ring distortion normal mode (figure f), characteristic of $\pi\pi_1$* relaxation. The CASPT2 spectral density of the Min-nπ* (white lines) locate intense vibrations at, respectively, 503 cm^{-1}(figure c), 526 cm^{-1} (figure d) and 691 cm^{-1} (figure e), addressable to the intense experimental energy modes.

In case of cadC, high noise of the extracted oscillation map makes it difficult to correctly identify the frequencies present in the measurement.

12. Measurement of pure solvent

Figure S18 shows the dynamics at selected probe energies of the TA measurement on the phosphate buffer solution. No signal from solvated electrons was observed under our experimental conditions. The signal visible at time zero is due to cross-phase modulation artifact.

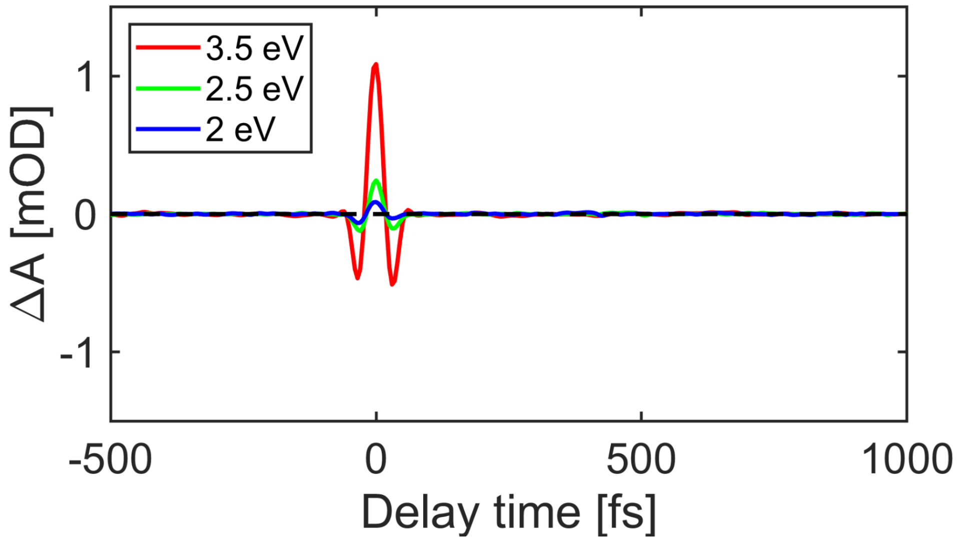

Figure S18: TA measurement on pure solvent showing no solvated electron signals.

13. Cartesian coordinates (QM region only)

a) 5-methyl-2'-deoxycytdine

Min S_0:

N	33.324679	32.859884	34.698988
C	32.933687	32.389114	33.430126
N	33.739849	32.683096	32.371342
C	34.919003	33.288765	32.558186
C	35.441590	33.614534	33.862437
C	34.574993	33.399460	34.902400
O	31.863488	31.745796	33.308698
N	35.639670	33.577800	31.460113
C	36.855239	34.091020	34.050318
H	34.852900	33.627269	35.934508
H	36.479458	34.173808	31.468562
H	35.233167	33.308114	30.557738
H	37.013091	35.103629	33.646645
H	37.117337	34.075944	35.117558
H	37.547351	33.409928	33.529125
H	32.692123	32.748789	35.485251

Plateau $\pi\pi_1$*:

N	33.390563	32.845746	34.762168
C	33.022947	32.359873	33.399139
N	33.817093	32.628686	32.367133
C	34.990751	33.358386	32.598795
C	35.523602	33.545783	33.909396
C	34.681775	33.236021	35.030802
O	31.939149	31.712588	33.363495
N	35.787068	33.522914	31.482180
C	36.928546	34.013194	34.124856
H	34.974774	33.419459	36.066109
H	36.456825	34.308172	31.439500
H	35.295394	33.332628	30.600389
H	37.059052	35.064338	33.811563
H	37.214265	33.899831	35.180179
H	37.615148	33.409035	33.509278
H	32.731368	32.730907	35.528919

Min $\pi\pi_1$*:

N	33.428646	32.712917	34.674383
C	33.137867	32.052847	33.398412
N	34.030111	32.175758	32.387196
C	35.021250	33.175846	32.501751
C	35.584924	33.341839	33.824402
C	34.733903	33.078288	34.919567
O	32.090485	31.361049	33.346696
N	35.855005	33.314614	31.373053
C	37.003607	33.770319	34.037029
H	35.045897	33.218194	35.957807
H	36.352268	34.224890	31.326335
H	35.318804	33.189907	30.499889
H	37.170706	34.788053	33.641003
H	37.269911	33.737674	35.103836
H	37.689940	33.100651	33.489465

H	32.768346	32.649939	35.444629

CI $\pi\pi_1^*/S_0$:

N	33.590051	32.635757	34.790263
C	33.378207	31.920943	33.520526
N	34.254484	32.066484	32.550325
C	34.953603	33.432827	32.594199
C	35.704883	33.121265	33.850394
C	34.908490	32.833482	34.989084
O	32.400513	31.121892	33.570363
N	35.830568	33.447675	31.451947
C	37.200999	33.151695	33.956548
H	35.310938	32.824657	36.007298
H	36.330725	34.354311	31.389786
H	35.269727	33.333611	30.592617
H	37.541823	34.143621	33.611918
H	37.553249	32.959676	34.977506
H	37.667575	32.415126	33.290785
H	32.911568	32.594765	35.542752

CI $\pi\pi_1^*/S_0$ ‘ethene-like’:

N	33.670529	32.790087	34.738734
C	33.351448	32.102763	33.368558
N	34.304949	32.123419	32.423027
C	35.451379	32.833976	32.633741
C	35.833192	33.160445	33.944238
C	34.984787	32.537157	35.014434
O	32.247885	31.537103	33.336627
N	36.110575	33.277006	31.493608
C	36.483502	34.480895	34.289313
H	35.333406	31.919190	35.851161
H	36.687196	34.129697	31.584207
H	35.504240	33.303628	30.660848
H	35.986929	34.936200	35.160473
H	37.537416	34.309104	34.569274
H	36.443598	35.191773	33.452565
H	32.962244	32.735973	35.469629

Min $n_N\pi^*$:

N	33.744821	32.555561	34.796601
C	33.514496	31.856668	33.608742
N	34.701555	31.725423	32.921532
C	35.569352	32.837630	32.780373
C	35.963345	33.333812	34.032786
C	35.049309	33.057723	35.067023
O	32.439559	31.294047	33.334632
N	36.008527	33.192278	31.507743
C	37.189777	34.186865	34.251115
H	35.250414	33.327342	36.104916
H	36.398853	34.151479	31.430820
H	35.293758	33.022747	30.783465
H	37.010219	35.253421	34.030194
H	37.530543	34.073993	35.292716
H	38.014479	33.860083	33.600463
H	33.022051	32.583029	35.508096

Min $^3n_O\pi^*$:

N	33.674326	32.795553	34.815844
C	33.504612	32.166805	33.613938
N	34.229960	32.296265	32.543895
C	35.361297	33.131857	32.648950
C	35.755455	33.651212	33.887336
C	34.908822	33.507870	34.997098
O	32.477664	31.295892	33.487512
N	36.145571	33.220374	31.480110
C	37.102050	34.313579	34.040417
H	35.166266	33.791849	36.014521
H	36.611460	34.142098	31.367694
H	35.564115	33.060438	30.643839
H	37.140927	35.300777	33.554110
H	37.353985	34.419382	35.103743
H	37.881279	33.689685	33.575740
H	33.011365	32.691157	35.575329

Min T_1:

N	33.777466	32.972429	34.647859
C	33.513296	32.343906	33.419352
N	34.338772	32.511824	32.328234
C	35.517217	33.149540	32.487065
C	35.912891	33.705287	33.717715
C	34.866167	33.839036	34.780687
O	32.488570	31.637166	33.307267
N	36.342346	33.181851	31.369659
C	37.254514	34.292921	33.984430
H	35.168518	34.079092	35.803753
H	36.860480	34.070893	31.235395
H	35.801788	32.948264	30.523025
H	37.184700	35.397734	34.007683
H	37.617460	33.947063	34.967183
H	37.988448	34.017703	33.218103
H	33.106465	32.868090	35.407052

CI $n_N\pi^*/S_0$:

N	33.768497	32.497253	34.970405
C	33.575577	31.834196	33.755097
N	34.897706	31.558299	33.370988
C	35.663382	32.787700	33.006827
C	36.021871	33.297224	34.218630
C	35.137806	32.799593	35.275064
O	32.532148	31.339834	33.311653
N	35.957136	33.075246	31.686364
C	37.103148	34.321117	34.483355
H	35.344845	32.939932	36.337496
H	36.298974	34.051491	31.566382
H	35.192501	32.867443	31.028515
H	36.769061	35.366264	34.352256
H	37.488333	34.182331	35.506943
H	37.946190	34.162860	33.795852
H	33.024091	32.572074	35.654066

CI $n_O\pi^*/\ \pi\pi_1^*$:

N	33.474468	32.716945	34.747971
C	33.178581	32.917392	33.447735
N	33.772651	33.236972	32.419429
C	35.280681	33.383635	32.663906
C	35.662887	33.579577	33.917786
C	34.686332	33.434535	35.018817
O	31.727132	32.614123	33.192353
N	36.052445	33.135702	31.519788
C	37.095211	33.976638	34.225948
H	34.779347	33.859953	36.017338
H	36.683937	33.954210	31.384514
H	35.434929	33.180391	30.695771
H	37.301736	35.000105	33.872572
H	37.324976	33.920179	35.298687
H	37.796122	33.295104	33.716793
H	32.778799	32.609795	35.496677

b) <u>5-hydroxymethyl-2’-deoxycytdine</u>

Min S_0:

N	34.400790	34.455786	34.556550
C	35.017301	34.198710	35.796393
N	34.411790	34.653620	36.931047
C	33.281137	35.373287	36.871593
C	32.637849	35.668286	35.617288
C	33.229004	35.163629	34.490526
O	36.103727	33.567841	35.848554
N	32.740022	35.782598	38.035814
C	31.388664	36.473157	35.535824
H	32.785517	35.309587	33.499555
H	31.963355	36.453891	38.065243
H	33.164619	35.493746	38.934090
O	31.761200	37.865714	35.743785
H	30.974287	38.359661	36.134711
H	30.914286	36.342757	34.548358
H	30.661546	36.169467	36.308138
H	34.826211	34.112037	33.699535

Plateau $\pi\pi_1$*:

N	34.365238	34.453839	34.529131
C	34.957610	34.176510	35.823105
N	34.382779	34.612901	36.972117
C	33.225391	35.406924	36.896828
C	32.587961	35.647123	35.651447
C	33.173426	35.154054	34.438248
O	36.035176	33.524387	35.815579
N	32.608703	35.710754	38.105769
C	31.320724	36.436802	35.587640
H	32.738882	35.319019	33.451031
H	32.077479	36.589714	38.147982
H	33.167617	35.544130	38.956462
O	31.670331	37.841142	35.768427
H	30.855449	38.325042	36.100182
H	30.819977	36.292239	34.616677
H	30.640239	36.122565	36.393416
H	34.813168	34.112215	33.682079

Min $\pi\pi_1$*:

N	34.398895	34.358308	34.484380
C	34.831871	33.809419	35.754177
N	34.222362	34.269036	36.875839
C	33.499084	35.488497	36.835891
C	32.689245	35.578877	35.635885
C	33.200197	35.024761	34.440911
O	35.668264	32.887031	35.732003
N	32.958644	35.842456	38.079899
C	31.356134	36.255588	35.657102
H	32.703168	35.151327	33.476035
H	32.562525	36.787887	38.114815
H	33.618131	35.691204	38.858421
O	31.631893	37.681244	35.756853

H	30.804501	38.152185	36.074878
H	30.770627	36.034825	34.747610
H	30.785063	35.929859	36.540803
H	34.829566	34.022089	33.628628

CI $\pi\pi_1$*/S_0:

N	34.525905	34.386626	34.403920
C	35.016267	33.879801	35.688426
N	34.534675	34.524884	36.763293
C	34.333842	35.993327	36.560900
C	33.125675	35.713903	35.738514
C	33.429525	35.141642	34.472760
O	35.703967	32.837626	35.657767
N	34.074986	36.644591	37.795906
C	31.759418	36.200740	36.101868
H	32.819734	35.289507	33.571950
H	33.703237	37.586328	37.630787
H	34.937884	36.712994	38.358960
O	31.865238	37.639614	35.900300
H	31.041180	38.091150	36.268684
H	30.967382	35.772452	35.468405
H	31.560289	35.986817	37.161805
H	34.889238	34.032936	33.527984

Min T_1:

N	34.014327	34.372520	34.545530
C	34.525571	34.009680	35.789787
N	33.878967	34.342692	36.971358
C	32.814414	35.182545	36.920850
C	32.224928	35.550540	35.697778
C	32.752803	34.962176	34.437620
O	35.614210	33.396372	35.873694
N	32.293371	35.569133	38.141191
C	31.076946	36.497801	35.624428
H	32.449674	35.343065	33.462905
H	31.835212	36.490701	38.172104
H	32.946385	35.429931	38.925983

O	31.616324	37.848900	35.736248
H	30.857595	38.434249	36.044955
H	30.548034	36.377941	34.666378
H	30.369862	36.312777	36.445209
H	34.514699	34.093011	33.706308

CI $n_N\pi^*/S_0$:

N	34.136255	34.504190	34.681114
C	34.715423	34.363125	35.908237
N	33.707181	34.225787	36.898146
C	32.887555	35.445651	36.999731
C	32.230486	35.625375	35.690878
C	32.838938	35.108522	34.591116
O	35.907668	34.209819	36.175809
N	33.570956	36.565259	37.574890
C	31.018245	36.490726	35.589610
H	32.419924	35.183396	33.588524
H	32.942033	37.375403	37.542462
H	33.788344	36.376386	38.565644
O	31.486181	37.867117	35.742629
H	30.707424	38.415191	36.077985
H	30.527470	36.352480	34.612251
H	30.308225	36.265546	36.397550
H	34.640742	34.250825	33.834994

CI $n_O\pi^*/\pi\pi_1^*$:

N	34.371655	34.522468	34.794802
C	34.863325	34.453269	36.025174
N	34.582579	34.957470	37.145136
C	33.292116	35.733489	37.056593
C	32.608193	35.808206	35.906415
C	33.188105	35.287580	34.646490
O	36.098199	33.650548	36.170338
N	33.004781	36.314957	38.278592

C	31.320309	36.563489	35.800735
H	32.590644	35.059081	33.762876
H	32.047631	36.684693	38.337529
H	33.223422	35.710624	39.091411
O	31.602908	37.997230	35.733114
H	30.786276	38.482009	36.041289
H	30.781598	36.254260	34.889803
H	30.673580	36.372000	36.670713
H	34.813694	34.126292	33.966988

c) <u>5-formyl-2'-deoxycytdine</u>

Min S_0:

N	33.945232	32.960624	36.941093
C	34.014868	32.482565	38.273395
N	34.820667	31.419849	38.539983
C	35.474330	30.764252	37.571193
C	35.419925	31.229898	36.202775
C	34.645159	32.341362	35.958813
O	33.334999	33.021800	39.172127
N	36.190044	29.700774	37.946264
C	36.102036	30.595114	35.067706
H	34.572261	32.754353	34.948238
H	36.594809	29.038140	37.269661
H	36.053461	29.340965	38.903125
O	35.945077	30.964707	33.890367
H	36.789624	29.758702	35.285082
H	33.344504	33.748935	36.713892

Plateau $\pi\pi_1$*:

N	33.931362	32.993988	36.849821
C	33.995148	32.501829	38.197176
N	34.830055	31.466228	38.513778
C	35.471416	30.805733	37.520437
C	35.431120	31.239944	36.148987
C	34.767705	32.517756	35.861683
O	33.269608	33.041329	39.067974
N	36.146068	29.703824	37.894071

C	35.898595	30.474708	35.030448
H	34.666849	32.904300	34.847895
H	36.739702	29.159638	37.251501
H	36.035641	29.360456	38.856962
O	35.813814	30.940715	33.846820
H	36.349083	29.483660	35.196484
H	33.300661	33.765354	36.634037

CI $\pi\pi_1^*/S_0$:

N	34.135990	32.951311	36.845863
C	33.976486	32.406983	38.196929
N	34.816731	31.414976	38.663135
C	35.607816	30.770110	37.787252
C	35.673030	31.293298	36.431645
C	35.396674	32.742778	36.319526
O	33.111283	32.894109	38.926302
N	36.172994	29.615453	38.128480
C	35.251804	30.416756	35.353339
H	36.090258	33.549012	36.068077
H	36.831616	29.150411	37.486364
H	35.939244	29.152949	39.018144
O	35.117277	30.825020	34.176785
H	35.139726	29.343024	35.595937
H	33.538713	33.733024	36.567803

Min $n_O\pi^*$-f:

N	33.959674	32.822292	36.822050
C	34.019347	32.379106	38.144131
N	34.849241	31.333922	38.470998
C	35.604245	30.738823	37.552703
C	35.686788	31.251987	36.186725
C	34.802844	32.296670	35.857790
O	33.310192	32.898012	39.034413
N	36.320248	29.671546	37.942780
C	36.643263	30.774221	35.279745
H	34.760566	32.725341	34.856591
H	36.813954	29.064670	37.278229

H	36.126858	29.288208	38.876416
O	36.751884	31.274736	34.037545
H	37.496512	30.140637	35.551746
H	33.332391	33.585845	36.584773

Min T_1:

N	34.025638	33.017698	36.853897
C	34.021316	32.484848	38.149413
N	34.863336	31.433233	38.476667
C	35.492296	30.761730	37.514653
C	35.482650	31.258126	36.136193
C	35.040073	32.665287	35.967915
O	33.267292	32.931573	39.029427
N	36.105683	29.617646	37.838382
C	35.813787	30.487253	34.950309
H	35.028888	33.115686	34.978279
H	36.729885	29.107847	37.199450
H	36.004551	29.264949	38.798106
O	35.787667	31.029725	33.826130
H	36.129049	29.436006	35.064696
H	33.385540	33.772075	36.621249

Min T_2:

N	33.872353	32.836701	36.923651
C	33.930451	32.386578	38.244384
N	34.773618	31.349565	38.568840
C	35.546323	30.777155	37.649412
C	35.634736	31.314983	36.297813
C	34.738765	32.337279	35.970973
O	33.209581	32.886719	39.134248
N	36.281315	29.716095	38.023369
C	36.605740	30.844196	35.372292
H	34.710247	32.778837	34.974673
H	36.777462	29.129407	37.341460
H	36.094133	29.307526	38.948309
O	36.754317	31.398559	34.182061
H	37.510055	30.300786	35.680807
H	33.245067	33.595440	36.674110

Min $n_O\pi^*$-c:

N	33.852802	32.973896	36.897447
C	33.744178	32.403365	38.199443
N	34.574184	31.376762	38.590820
C	35.176885	30.646745	37.648102
C	35.150726	31.069179	36.251135
C	34.510979	32.261982	35.955622
O	33.646007	33.381253	39.208238
N	35.818693	29.515997	38.006565
C	35.710247	30.294888	35.151735
H	34.484895	32.633311	34.929139
H	36.524017	29.085770	37.396515
H	35.828288	29.237130	38.992594
O	35.798749	30.734329	33.989205
H	36.035225	29.262392	35.364910
H	33.285261	33.775505	36.639897

Min $^3n_O\pi^*$-c:

N	33.808627	32.922756	36.870372
C	33.677900	32.345048	38.170674
N	34.491481	31.291534	38.545947
C	35.095550	30.568342	37.599564
C	35.096930	31.014575	36.209486
C	34.478275	32.222875	35.927475
O	33.651089	33.327958	39.172923
N	35.726780	29.428172	37.953280
C	35.678227	30.261828	35.106031
H	34.472797	32.614641	34.908403
H	36.459412	29.022725	37.358920
H	35.740990	29.158284	38.941986
O	35.788141	30.723318	33.954004
H	36.003972	29.227141	35.307948
H	33.254917	33.735618	36.618061

CI $n_O\pi^*$-f/S_0:

N	33.898706	32.853987	36.846175

C	33.983765	32.417979	38.146922
N	34.814636	31.346625	38.500152
C	35.541609	30.712343	37.608623
C	35.637161	31.181599	36.199259
C	34.720056	32.313187	35.813001
O	33.296274	32.916773	39.065524
N	36.240505	29.631875	37.998051
C	36.514449	30.618076	35.446472
H	34.643547	32.734629	34.816723
H	36.693763	29.005701	37.324779
H	36.063380	29.259513	38.939189
O	36.720616	31.195523	33.827512
H	37.407291	30.021363	35.601148
H	33.273498	33.623482	36.621480

CI $n_O\pi^*$-c/S_0:

N	33.940571	33.090387	36.864829
C	33.957715	32.599799	38.214203
N	34.272847	31.281074	38.480091
C	35.102151	30.653589	37.542749
C	35.150905	31.139912	36.206197
C	34.477455	32.338645	35.909760
O	35.061300	32.963978	39.034123
N	35.812987	29.626860	37.989573
C	35.729870	30.379769	35.100600
H	34.352263	32.642648	34.868429
H	36.530176	29.160471	37.417891
H	35.685040	29.294536	38.951779
O	35.834275	30.830423	33.948115
H	36.063925	29.348877	35.309775
H	33.362591	33.890461	36.635794

d) <u>5-carboxyl-2’-deoxycytdine</u>

Min S_0:

N	33.764622	33.105071	36.853166
C	33.761522	32.361384	38.049565
N	32.979198	32.789487	39.073955
C	32.232338	33.911307	38.956316

C	32.318545	34.761341	37.789195
C	33.083646	34.286099	36.756933
O	34.463170	31.314458	38.110385
N	31.378251	34.191948	39.945733
C	31.573241	36.056226	37.611634
H	33.151392	34.841016	35.820373
H	30.774956	35.010447	39.815421
H	31.235616	33.518682	40.716118
O	31.749144	36.710272	36.528401
O	30.776254	36.407408	38.541278
H	34.259533	32.739976	36.045242

Plateau $\pi\pi_1$*:

N	33.802858	33.122066	36.828009
C	33.764897	32.337347	38.061365
N	33.008371	32.764911	39.096855
C	32.246900	33.901701	38.935518
C	32.347003	34.777649	37.815352
C	33.286268	34.398261	36.735463
O	34.453133	31.281087	38.056234
N	31.365518	34.159278	39.941439
C	31.554322	36.022788	37.656800
H	33.359532	34.947776	35.799290
H	30.750988	34.972762	39.813915
H	31.211182	33.461068	40.686076
O	31.749342	36.703853	36.583898
O	30.717814	36.348601	38.567719
H	34.294713	32.730373	36.026436

CI $\pi\pi_1$*/S_0:

N	33.663396	33.291282	36.939442
C	33.676663	32.450922	38.175291
N	33.112739	32.924606	39.313159
C	32.414029	34.093357	39.261645
C	32.693887	34.977360	38.173886
C	33.883758	34.582178	37.385288
O	34.276984	31.359159	38.090834

N	31.368014	34.214155	40.107040
C	31.628902	35.762135	37.486771
H	34.831885	35.109611	37.279992
H	30.834359	35.084247	40.129732
H	31.182358	33.464076	40.792521
O	31.743609	35.994934	36.234683
O	30.710148	36.230513	38.241294
H	34.163338	32.927466	36.130358

References

(1) D.A. Case, T.A. Darden, T.E. Cheatham, III, C.L. Simmerling, J. Wang, R.E. Duke, R.; Luo, R.C. Walker, W. Zhang, K.M. Merz, B. Roberts, S. Hayik, A. Roitberg, G. S.; J. Swails, A.W. Götz, I. Kolossváry, K.F. Wong, F. Paesani, J. Vanicek, R.M. Wolf, J. L.; X. Wu, S.R. Brozell, T. Steinbrecher, H. Gohlke, Q. Cai, X. Ye, J. Wang, M.-J. Hsieh, G.; Cui, D.R. Roe, D.H. Mathews, M.G. Seetin, R. Salomon-Ferrer, C. Sagui, V. Babin, T.; Luchko, S. Gusarov, A. Kovalenko, and P. A. K. AMBER 2012. Univeristy of California, San Francisco 2012.

(2) Jakalian, A.; Jack, D. B.; Bayly, C. I. Fast, Efficient Generation of High-Quality Atomic Charges. AM1-BCC Model: II. Parameterization and Validation. *J. Comput. Chem.* **2002**, *23*, 1623–1641.

(3) Wang, J.; Wolf, R. M.; Caldwell, J. W.; Kollman, P. A.; Case, D. A. Development and Testing of a General Amber Force Field. *J. Comput. Chem.* **2004**, *25*, 1157–1174.

(4) Daszykowski, M.; Walczak, B. Density-Based Clustering Methods. *Compr. Chemom.* **2009**, *2*, 635–654.

(5) Watanabe, K.; Kawai, G.; Ueda, T. Conformational Properties of a Novel Modified Nucleoside, 5-Formylcytidine, Found at the First Position of the Anticodon of Bovine Mitochondrial Trnamet. *Nucleosides and Nucleotides* **1994**, *13*, 1189–1199.

(6) Mahto, S. K.; Chow, C. S. Synthesis and Solution Conformation Studies of the Modified Nucleoside N4,2′-O-Dimethylcytidine (M4Cm) and Its Analogues. *Bioorganic Med. Chem.* **2008**, *16*, 8795–8800.

(7) Szulik, M. W.; Pallan, P. S.; Nocek, B.; Voehler, M.; Banerjee, S.; Brooks, S.; Joachimiak, A.; Egli, M.; Eichman, B. F.; Stone, M. P. Differential Stabilities and Sequence-Dependent Base Pair Opening Dynamics of Watson-Crick Base Pairs with 5-Hydroxymethylcytosine, 5-Formylcytosine, or 5-Carboxylcytosine. *Biochemistry* **2015**, *54*, 1294–1305.

(8) Weingart, O.; Nenov, A.; Altoè, P.; Rivalta, I.; Segarra-Martí, J.; Dokukina, I.; Garavelli, M. COBRAMM 2.0 — A Software Interface for Tailoring Molecular Electronic Structure Calculations and Running Nanoscale (QM/MM) Simulations. *J. Mol. Model.* **2018**, *24*, 271.

(9) Aquilante, F.; Autschbach, J.; Carlson, R. K.; Chibotaru, L. F.; Delcey, M. G.; De Vico, L.; Fdez. Galván, I.; Ferré, N.; Frutos, L. M.; Gagliardi, L.; Garavelli, M.; Giussani, A.; Hoyer, C. E.; Li Manni, G.; Lischka, H.; Ma, D.; Malmqvist, P. Å.; Müller, T.; Nenov, A.; Olivucci, M.; Pedersen, T. B.; Peng, D.; Plasser, F.; Pritchard, B.; Reiher, M.; Rivalta, I.; Schapiro, I.; Segarra-Martí, J.; Stenrup, M.; Truhlar, D. G.; Ungur, L.; Valentini, A.; Vancoillie, S.; Veryazov, V.; Vysotskiy, V. P.; Weingart, O.; Zapata, F.; Lindh, R. Molcas 8: New Capabilities for Multiconfigurational Quantum Chemical Calculations across the Periodic Table. *J. Comput. Chem.* **2016**, *37*, 506–541.

(10) Fdez. Galván, I.; Vacher, M.; Alavi, A.; Angeli, C.; Aquilante, F.; Autschbach, J.; Bao, J. J.; Bokarev, S. I.; Bogdanov, N. A.; Carlson, R. K.; Chibotaru, L. F.; Creutzberg, J.; Dattani, N.; Delcey, M. G.; Dong, S. S.; Dreuw, A.; Freitag, L.; Frutos, L. M.; Gagliardi, L.; Gendron, F.; Giussani, A.; González, L.; Grell, G.; Guo, M.; Hoyer, C. E.; Johansson, M.; Keller, S.; Knecht, S.; Kovačević, G.; Källman, E.; Li Manni, G.; Lundberg, M.; Ma, Y.; Mai, S.; Malhado, J. P.; Malmqvist, P. Å.; Marquetand, P.; Mewes, S. A.; Norell, J.; Olivucci, M.; Oppel, M.; Phung, Q. M.; Pierloot, K.; Plasser, F.; Reiher, M.; Sand, A. M.; Schapiro, I.; Sharma, P.; Stein, C. J.; Sørensen, L. K.; Truhlar, D. G.; Ugandi, M.; Ungur, L.; Valentini, A.; Vancoillie, S.; Veryazov, V.; Weser, O.; Wesołowski, T. A.; Widmark, P.-O.; Wouters, S.; Zech, A.; Zobel, J. P.; Lindh, R. OpenMolcas: From Source Code to Insight. *J. Chem. Theory Comput.* **2019**, *15*, 5925–5964.

(11) Aquilante, F.; Autschbach, J.; Baiardi, A.; Battaglia, S.; Borin, V. A.; Chibotaru, L. F.; Conti, I.; De Vico, L.; Delcey, M.; Fdez. Galván, I.; Ferré, N.; Freitag, L.; Garavelli, M.; Gong, X.; Knecht, S.; Larsson, E. D.; Lindh, R.; Lundberg, M.; Malmqvist, P. Å.; Nenov, A.; Norell, J.; Odelius, M.; Olivucci, M.; Pedersen, T. B.; Pedraza-González, L.; Phung, Q. M.; Pierloot, K.; Reiher, M.; Schapiro, I.; Segarra-Martí, J.; Segatta, F.; Seijo, L.; Sen, S.; Sergentu, D.-C.; Stein, C. J.; Ungur, L.; Vacher, M.; Valentini, A.; Veryazov, V. Modern Quantum Chemistry with [Open]Molcas. *J. Chem. Phys.* **2020**, *152*, 214117.

(12) Frisch, M. J.; Trucks, G. W.; Schlegel, H. B.; Scuseria, G. E.; Robb, M. A.; Cheeseman, J. R.; Scalmani, G.; Barone, V.; Petersson, G. A.; Nakatsuji, H.; Li, X.; Caricato, M.; Marenich, A. V; Bloino, J.; Janesko, B. G.; Gomperts, R.; Mennucci, B.; Hratchian, H. P.; Ortiz, J. V; Izmaylov, A. F.; Sonnenberg, J. L.; Williams-Young, D.; Ding, F.; Lipparini, F.; Egidi, F.; Goings, J.; Peng, B.; Petrone, A.; Henderson, T.; Ranasinghe, D.; Zakrzewski, V. G.; Gao, J.; Rega, N.; Zheng, G.; Liang, W.; Hada, M.; Ehara, M.; Toyota, K.; Fukuda, R.; Hasegawa, J.; Ishida, M.; Nakajima, T.; Honda, Y.; Kitao, O.; Nakai, H.; Vreven, T.; Throssell, K.; Montgomery Jr., J. A.; Peralta, J. E.; Ogliaro, F.; Bearpark, M. J.; Heyd, J. J.; Brothers, E. N.; Kudin, K. N.; Staroverov, V. N.; Keith, T. A.; Kobayashi, R.; Normand, J.; Raghavachari, K.; Rendell, A. P.; Burant, J. C.; Iyengar, S. S.; Tomasi, J.; Cossi, M.; Millam, J. M.; Klene, M.; Adamo, C.; Cammi, R.; Ochterski, J. W.; Martin, R. L.; Morokuma, K.; Farkas, O.; Foresman, J. B.; Fox, D. J. Gaussian˜16 {R}evision {C}.01. 2016.

(13) Zobel, J. P.; Nogueira, J. J.; González, L. The IPEA Dilemma in CASPT2. *Chem. Sci.* **2017**, *8*, 1482–1499.

(14) Forsberg, N.; Malmqvist, P.-Å. Multiconfiguration Perturbation Theory with Imaginary Level Shift. *Chem. Phys. Lett.* **1997**, *274*, 196–204.

(15) Bearpark, M. J.; Larkin, S. M.; Vreven, T. Searching for Conical Intersections of Potential Energy Surfaces with the ONIOM Method: Application to Previtamin D. *J. Phys. Chem. A* **2008**, *112*, 7286–7295.

(16) Martínez-Fernández, L.; Pepino, A. J.; Segarra-Martí, J.; Jovaišaitė, J.; Vaya, I.; Nenov, A.; Markovitsi, D.; Gustavsson, T.; Banyasz, A.; Garavelli, M.; Improta, R. Photophysics of Deoxycytidine and 5-Methyldeoxycytidine in Solution: A Comprehensive Picture by Quantum Mechanical Calculations and Femtosecond Fluorescence Spectroscopy. *J. Am. Chem. Soc.* **2017**, *139*, 7780–7791.

(17) Wang, X.; Zhou, Z.; Tang, Y.; Chen, J.; Zhong, D.; Xu, J. Excited State Decay Pathways of 2′-Deoxy-5-Methylcytidine and Deoxycytidine Revisited in Solution: A Comprehensive Kinetic Study by Femtosecond Transient Absorption. *J. Phys. Chem. B* **2018**, *122*, 7027–7037.

(18) Wang, X.; Martínez-Fernández, L.; Zhang, Y.; Zhang, K.; Improta, R.; Kohler, B.; Xu, J.; Chen, J. Solvent-Dependent Stabilization of a Charge Transfer State Is the Key to Ultrafast Triplet State Formation in an Epigenetic DNA Nucleoside. *Chem. – A Eur. J.* **2021**, *27*, 1–10.

(19) Münzel, M.; Globisch, D.; Trindler, C.; Carell, T. Efficient Synthesis of 5-Hydroxymethylcytosine Containing DNA. *Org. Lett.* **2010**, *12*, 5671–5673.

(20) Münzel, M.; Lischke, U.; Stathis, D.; Pfaffeneder, T.; Gnerlich, F. A.; Deiml, C. A.; Koch, S. C.; Karaghiosoff, K.; Carell, T. Improved Synthesis and Mutagenicity of Oligonucleotides Containing 5-Hydroxymethylcytosine, 5-Formylcytosine and 5-Carboxylcytosine. *Chem. – A Eur. J.* **2011**, *17*,

13782–13788.

(21) Borrego-Varillas, R.; Ganzer, L.; Cerullo, G.; Manzoni, C. Ultraviolet Transient Absorption Spectrometer with Sub-20-Fs Time Resolution. *Applied Sciences* . **2018**, 8,989.

(22) Borrego-Varillas, R.; Oriana, A.; Branchi, F.; De Silvestri, S.; Cerullo, G.; Manzoni, C. Optimized Ancillae Generation for Ultra-Broadband Two-Dimensional Spectral-Shearing Interferometry. *J. Opt. Soc. Am. B* **2015**, *32*, 1851–1855.
